# An Engineered Biomimetic Nanoreactor With Cascade Functions for Targeted and Combinatorial Therapy of Ischemic Stroke

**DOI:** 10.1002/adhm.71712

**Published:** 2026-09-13

**Authors:** Shaofa Li, Ying Wu, Huimin Wei, Jinyong Teng, Zaizhi Yang, Jufang Chen, Bao Liao, Xiaoqin Huang, Lanqing Meng, Tong Li

**Affiliations:** ^1^ Youjiang Medical University For Nationalities Affiliated Southwest Hospital Baise People's Hospital Guangxi China; ^2^ Department of Neurology Affiliated Hospital of Youjiang Medical University for Nationalities Guangxi China; ^3^ Nanning Second People's Hospital Neurology Department Guangxi China

**Keywords:** antioxidant, biomimetic nanomedicine, ischemic stroke, neuroprotection, prussian blue nanoparticles, reperfusion injury, targeted delivery

## Abstract

Ischemic stroke reperfusion injury is driven by oxidative stress and neuroinflammation, but current neuroprotective strategies suffer from poor targeting, limited functionality, and low blood‑‐brain barrier (BBB) penetration. Herein, a multifunctional nanoplatform (RM@HPAN) is constructed: a hollow mesoporous Prussian blue core with multi‑enzyme mimetic activity, loaded with L‑arginine and butylphthalide, cloaked with red blood cell membrane for immune evasion, and modified with RVG‑29 for brain targeting. RM@HPAN exhibits ideal nanosize, pH‑responsive release, and robust ROS scavenging. It crosses a BBB model in vitro and boosts neuronal viability under oxygen‑‐glucose deprivation/reoxygenation. In a mouse middle cerebral artery occlusion model, RM@HPAN targets ischemic regions, reduces infarct volume from 29.4% to 3.8%, improves neurological scores, and rebalances inflammatory cytokines (TNF‑α, IL‑6, IL‑10, TGF‑β1). Transcriptomic and proteomic analyses reveal that RM@HPAN suppresses cytokine‑cytokine receptor interaction and TNF signaling while activating Rap1/PI3K/Akt and Nrf2/HO‑1 pathways, thereby inhibiting apoptosis and oxidative stress. Biosafety assessments confirm good biocompatibility. Thus, RM@HPAN offers a promising translatable strategy for ischemic stroke.

## Graphical abstract

Preparation of RVG‐modified erythrocyte membrane‐coated HMPB nanocarriers loaded with L‐arginine and NBP (RM@HPAN) and its neuroprotective mechanism. After intravenous injection, RM@HPAN achieves prolonged circulation, RVG‐29‐mediated BBB penetration, and ischemic accumulation. It exerts synergistic effects: ROS scavenging, NO bioavailability maintenance, anti‐inflammatory/anti‐apoptotic signaling, and endogenous antioxidant induction.

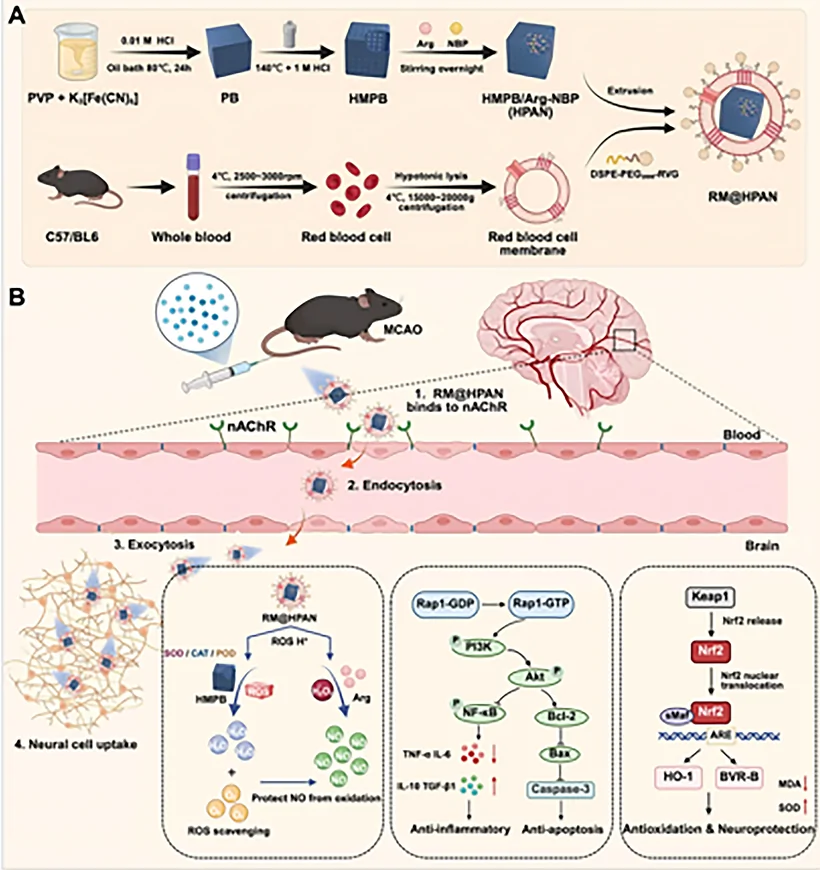

## 1 Introduction

Ischemic stroke, primarily caused by cerebral artery occlusion, remains one of the leading causes of death and long‐term disability worldwide [1, 2, 3]. Although reperfusion therapies such as intravenous thrombolysis and endovascular thrombectomy can restore blood flow, the subsequent ischemia‐reperfusion injury often counteracts the benefits of reperfusion and may even exacerbate neurological deficits [4, 5]. This injury process involves a complex pathophysiological cascade: the burst of reactive oxygen species (ROS) during early reperfusion triggers oxidative stress, which then activates multiple inflammatory pathways, leading to disruption of blood‐brain barrier (BBB) integrity and ultimately resulting in neuronal apoptosis and necrosis [6, 7]. Therefore, an ideal future neuroprotective strategy must possess multiple synergistic functions: the ability to efficiently cross the intact BBB [8], deliver therapeutics precisely to the ischemic penumbra [9], effectively alleviate oxidative stress and neuroinflammation, and promote the survival and repair of damaged neurons [10].

In recent years, nanomedicine has opened new avenues to address these challenges [11, 12]. Among various nanomaterials, Prussian blue nanoparticles have attracted significant attention due to their favorable biocompatibility, controllable synthesis [13], and particularly their remarkable multi‑enzyme mimetic antioxidant activity. They can simultaneously mimic superoxide dismutase, catalase, and peroxidase, efficiently scavenging multiple ROS such as superoxide anions and hydrogen peroxide, directly counteracting the core oxidative stress component in ischemia‑reperfusion injury [14, 15].

However, unmodified Prussian blue nanoparticles face major in vivo challenges: they are readily recognized and rapidly cleared by the mononuclear phagocyte system, resulting in a short circulatory half‑life [16]; they inherently lack specificity for brain tissue, making it difficult for them to effectively cross the BBB [17]; and their limited drug‑loading capacity hinders clinical translation [18]. To overcome these bottlenecks, biomimetic nanotechnology offers innovative solutions. Encapsulation within natural red blood cell membranes (RCM) endows nanoparticles with “self‑recognition” markers such as CD47, leveraging the CD47‐SIRPα “don't eat me” signaling pathway to achieve prolonged systemic circulation and immune evasion [19]. Further surface modification with the brain‑targeting peptide RVG‑29 enables specific recognition of nicotinic acetylcholine receptors (nAChRs) highly expressed on brain microvascular endothelial cells, actively mediating transport across the BBB and guiding accumulation in ischemic regions [20, 21].

Based on these concepts, we rationally designed an integrated multifunctional nanoplatform termed RM@HPAN (Scheme 1). The system has a hierarchical structure: a hollow mesoporous Prussian blue (HMPB) core for high‑efficiency co‑loading of two neuroprotective agents—L‑arginine (Arg, a nitric oxide precursor) and Dl‑3‑n‑butylphthalide (NBP, a clinical anti‑ischemic drug) [22, 23]; an outer red blood cell membrane cloak to confer long circulation and macrophage escape; and a surface‐anchored RVG‐29 peptide to mediate active brain targeting. This design synergistically combines the advantages of each component, aiming to achieve extended blood residence, efficient BBB crossing and ischemic site accumulation, on‐demand drug release in the acidic ischemic microenvironment, and combined effects of ROS scavenging, anti‐inflammation, anti‐apoptosis, and neuroprotection.

**SCHEME 1:**
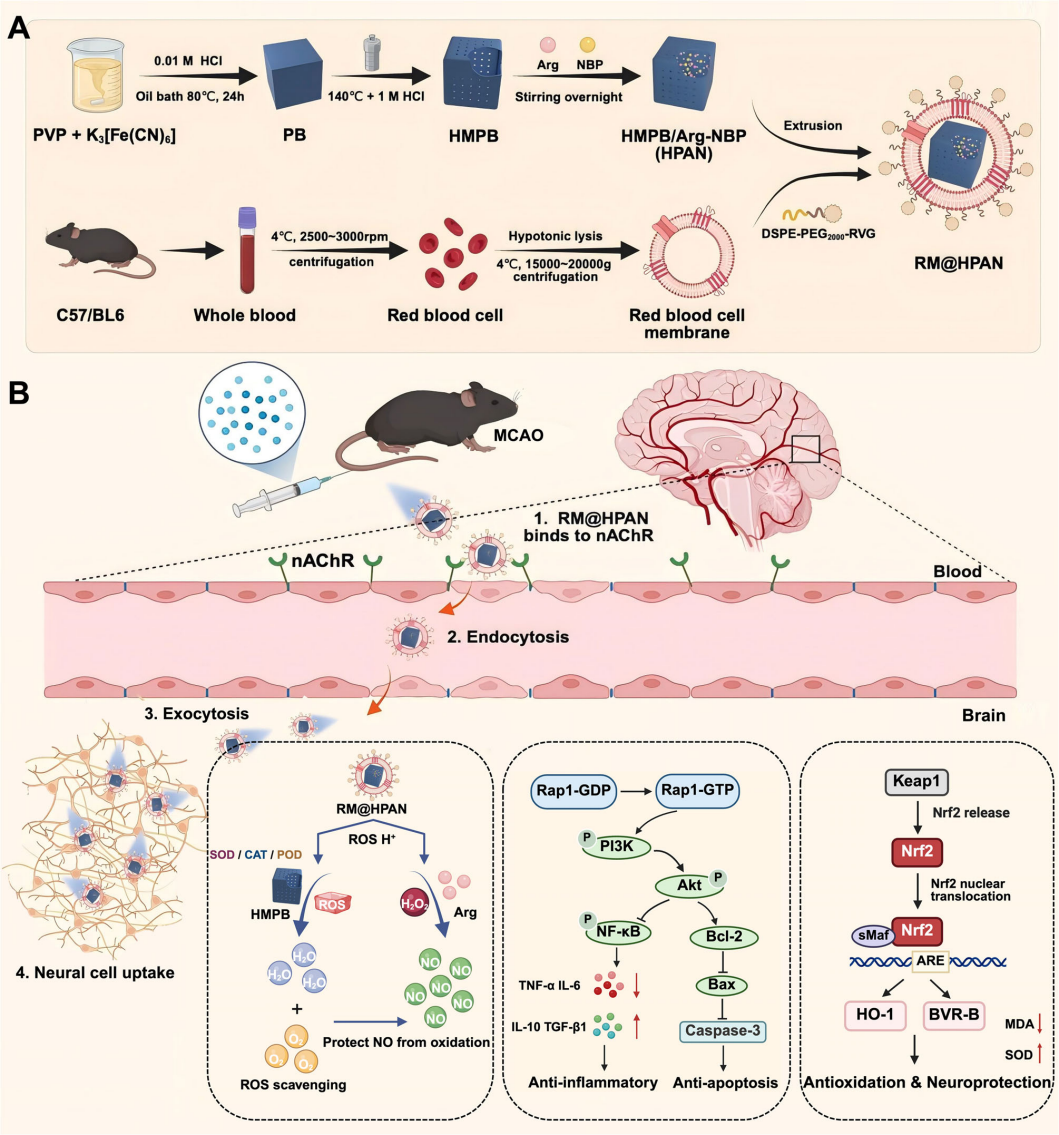
Preparation of RM@HPAN and its synergistic neuroprotective mechanism in the treatment of ischemic stroke. (A) Preparation process of RVG‐modified erythrocyte membrane‐coated HMPB nanocarriers loaded with L‐arginine and NBP (RM@HPAN). (B) In vivo blood–brain barrier penetration and delivery process of RM@HPAN, and its multi‐pathway synergistic neuroprotective mechanism against cerebral ischemic injury in the MCAO model. Following intravenous administration, the erythrocyte membrane‐coated structure prolongs the in vivo circulation time of the nanomaterial; subsequently, RM@HPAN penetrates the blood–brain barrier via RVG‐29‐mediated active targeting and accumulates at the ischemic site, exerting multiple synergistic therapeutic effects: including nanoenzyme‐mediated scavenging of excess reactive oxygen species, maintenance of nitric oxide bioavailability, activation of anti‐inflammatory and anti‐apoptotic signaling pathways, and induction of endogenous antioxidant stress responses (This image was created using BioRender (2026) https://BioRender.com).

We hypothesize that RM@HPAN can effectively navigate the pathological landscape of ischemic stroke. The RVG‐29 peptide guides its accumulation in the ischemic brain vasculature, while the red blood cell membrane enables evasion of immune clearance. Once internalized by compromised cells in the acidic ischemic milieu, the HMPB core degrades, releasing Arg and NBP. Concurrently, the HMPB core and the released agents work in concert to neutralize ROS, suppress inflammatory signaling, inhibit apoptotic pathways, and support neuronal repair. This manuscript details the systematic fabrication, comprehensive characterization, and multi‐level evaluation of RM@HPAN, encompassing its physicochemical properties, in vitro neuroprotective efficacy, in vivo targeting and biodistribution, therapeutic outcomes in a middle cerebral artery occlusion (MCAO) mouse model, underlying molecular mechanisms revealed by transcriptomics and proteomics, and preliminary biosafety assessment, collectively establishing its potential as a novel and potent nanotherapeutic candidate for ischemic stroke.

## 2 Results and Discussion

### 2.1 Preparation and Characterization of RM@HPAN

In this study, we designed and successfully constructed a composite nanosystem, RM@HPAN. This system is based on HMPB nanoparticles co‐loaded with Arg and NBP, sequentially coated with a red blood cell membrane and surface‐modified with DSPE‐PEG_2000_‐RVG. It aims to achieve multiple objectives: prolonging in vivo circulation, efficiently penetrating the BBB, and synergistically enhancing antioxidant and anti‐inflammatory activities for the treatment of middle cerebral artery occlusion models. First, Prussian blue nanoparticles (PB) were synthesized via a hydrothermal method [24]. Subsequently, under the protection of polyvinylpyrrolidone (PVP), they were etched at 140°C for 4 h to successfully prepare HMPB with a hollow mesoporous structure (Figure 1A). Nitrogen adsorption–desorption (BET) analysis revealed that HMPB has a specific surface area of 135.095 m^2^/g and a pore size of 20.167 nm (Figure 1J). Its high surface area and mesoporous characteristics provide an ideal foundation for efficient drug loading and controlled release. X‐ray diffraction (XRD) patterns indicated that HMPB maintains a face‐centered cubic crystal structure. Its diffraction peaks, corresponding to crystal planes (200), (220), (400), (420), (422), (440), (600), and (620), completely match the standard card for Prussian blue (space group Fm3m) (Figure 1K) [25]. This confirms that the etching process did not alter its crystal structure. Transmission electron microscopy (TEM) images showed that HPAN, after dual‐drug loading, retains a cubic morphology. The noticeably darker central region of the particles compared to HMPB suggests successful drug loading within the mesoporous cavities (Figure 1B). UV–Vis absorption spectra showed that PB, HMPB, and HPAN all exhibit the characteristic absorption peak of Prussian blue near 710 nm. Simultaneously, HPAN displayed characteristic peaks of Arg and NBP at 210 nm and 274 nm, respectively, confirming the successful dual–drug loading (Figure 1H) [26, 27, 28]. Fourier‐transform infrared spectroscopy (FTIR) analysis further verified the structural integrity of HPAN: the absorption peak at 2090 cm^−1^ is attributed to the stretching vibration of the Fe–CN–Fe bond in HMPB, while the peak at 1670 cm^−1^ corresponds to the C═O stretching vibration of the amide unit in PVP (Figure 1I) [29].

**FIGURE 1:**
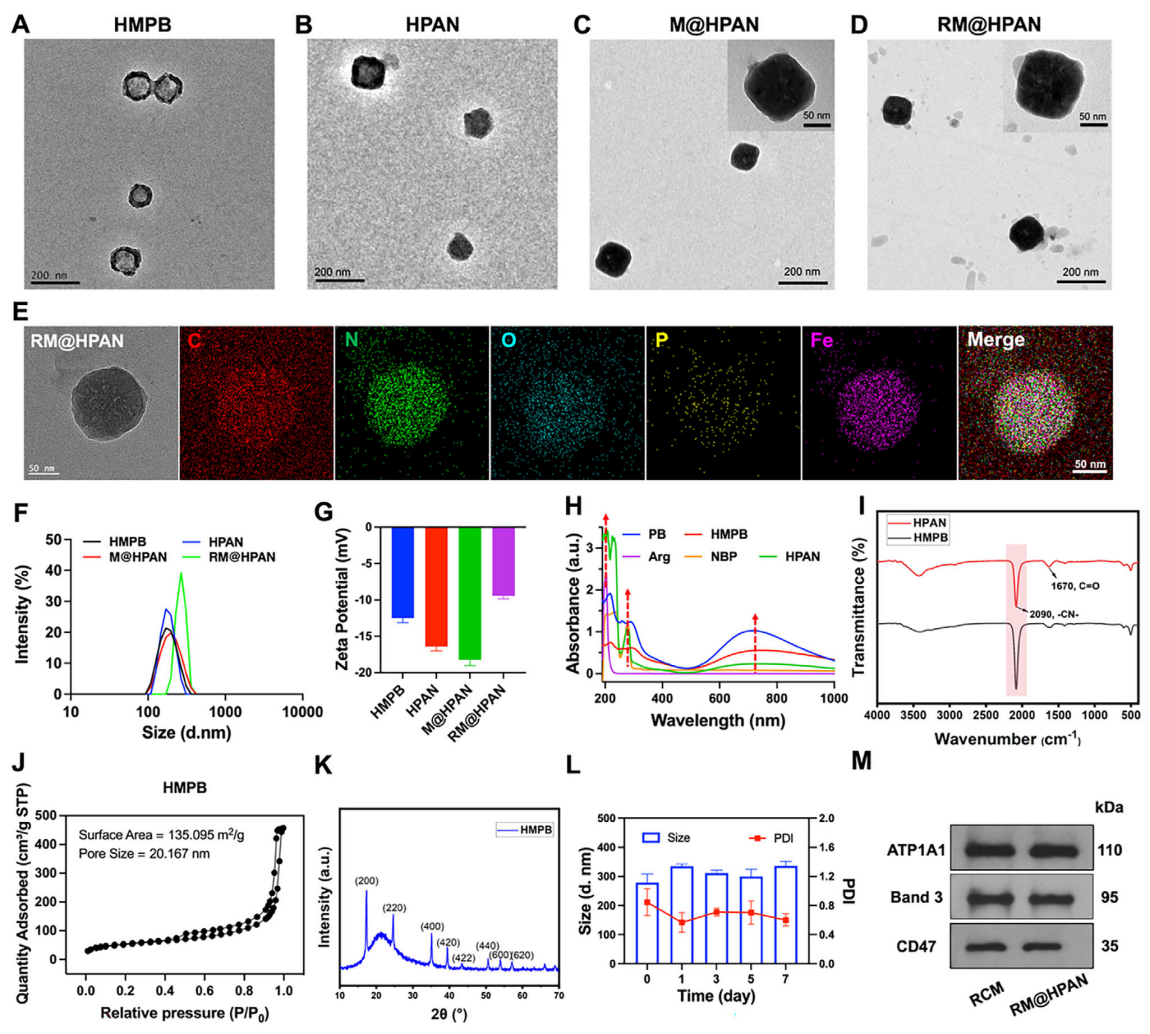
Preparation and characterization of RM@HPAN. TEM images of (A) HMPB; (B) HPAN; (C) M@HPAN; and (D) RM@HPAN. (E) Elemental mapping of RM@HPAN. (F) Particle size distribution of HMPB, HPAN, M@HPAN, RM@HPAN. (G) Zeta potentials of HMPB, HPAN, M@HPAN, RM@HPAN. (H) UV−vis spectroscopy of PB, HMPB, Arg, NBP, and HPAN. (I) FTIR spectroscopy of HMPB, and HPAN. (J) The isotherm adsorption–desorption curve of HMPB. (K) XRD patterns of HMPB. (L) Variation in mean particle sizes of RM@HPAN in PBS solution. (M) Western blot analysis of CD47 and Band 3 protein of different groups. Data were expressed as mean ± SD (*n* = 3).

TEM images revealed that both M@HPAN and RM@HPAN exhibit distinct core‐shell bilayer structures, confirming the successful coating of the red blood cell membrane onto the HPAN surface (Figure 1C,D). The elemental mapping of RM@HPAN further displayed signals for carbon (C), nitrogen (N), oxygen (O), and iron (Fe). The presence of oxygen may be attributed to the loading of Arg and NBP, as well as the modification with DSPE‐PEG_2000_‐RVG, while the phosphorus (P) signal suggests the presence of red blood cell membrane components (Figure 1E). Dynamic light scattering analysis indicated that the hydrodynamic diameters of the nanoparticles progressively increased with successive modifications: HMPB measured 170.6 ± 12.0 nm, HPAN 183.6 ± 9.7 nm, M@HPAN 210 ± 1.5 nm, and RM@HPAN 243.9 ± 1.2 nm (Figure 1F). Zeta potential measurements yielded the following results: HMPB at −13.6 ± 1.1 mV, HPAN at −17.0 ± 1.3 mV, M@HPAN at −18.1 ± 2.1 mV, and RM@HPAN at −9.4 ± 0.4 mV. The further negative shift in the potential of M@HPAN is likely due to the coating with the negatively charged red blood cell membrane, while the shift of RM@HPAN toward electrical neutrality may be attributed to the surface modification with the positively charged RVG‐29 peptide (Figure 1G). The colloidal stability of RM@HPAN was further monitored over 7 days (Figure 1L). The hydrodynamic diameter remained between 280 nm and 330 nm, with polydispersity index (PDI) values ranging from 0.65 to 0.85, indicating that no significant aggregation occurred during this period.

SDS‐PAGE analysis showed that the protein band patterns of M@HPAN and RM@HPAN were highly consistent with those of natural RCM, demonstrating that the red blood cell membranes were completely coated during the preparation process and their intrinsic protein composition remained unchanged (Figure S2). Western blot analysis further confirmed that CD47, a key membrane protein mediating immune escape, was successfully transferred to the surface of RM@HPAN. Additionally, we supplemented the detection of Band 3, another characteristic protein of red blood cell membranes, and the results proved that RM@HPAN retained the same protein bands as pure red blood cell membranes (Figure 1M). Furthermore, fluorescence colocalization experiments (Figure S3) revealed that the membrane fluorescence signal of DiO was highly colocalized with the fluorescence signal of the nanoparticle core, verifying that the red blood cell membranes were completely and stably coated on the surface of the nanoparticle core. Collectively, through the above multi‐dimensional characterization methods, we fully confirmed the successful coating of red blood cell membranes on the nanoparticles.

Quantification via a UV spectrophotometric standard curve determined that the encapsulation efficiency and drug loading capacity for Arg in HPAN were 42.8 ± 1.2% and 21.4 ± 0.6%, respectively, while for NBP, the values were 55.4 ± 0.8% and 2.8 ± 0.04% (Table S1). The high drug loading capacity can be attributed to the hollow mesoporous structure of HMPB. Drug release experiments (Figure S4) revealed that both HPAN and RM@HPAN exhibited rapid release behavior in an acidic environment (pH 5.0). The cumulative release rates for Arg and NBP reached 97.3 ± 3.0%–81.3 ± 2.6% and 94.3 ± 5.5%–78.5 ± 4.3%, respectively. In contrast, under neutral conditions (pH 7.4), the release was slower, with cumulative release rates of 69.6 ± 3.6%–51.7 ± 2.3% for Arg and 56.3 ± 3.1%–39.3 ± 3.2% for NBP. The accelerated release in acidic conditions is likely due to the weakened carrier‐drug interactions, a pH‐responsive characteristic beneficial for achieving precise and controlled drug release within the acidic microenvironment of cerebral ischemia. Furthermore, the red blood cell membrane coating effectively mitigated the initial burst release of the drugs, resulting in a more stable and controllable release profile for both Arg and NBP.

### 2.2 Synergistic NO Generation and Multienzyme‐Mimetic ROS‐Scavenging Properties of RM@HPAN

During reperfusion, excessive reactive oxygen species (ROS), including superoxide anion (O_2_•^−^), hydrogen peroxide (H_2_O_2_), and hydroxyl radical (•OH), trigger severe oxidative stress and neuroinflammation, which further disrupt the BBB and induce neuronal apoptosis, ultimately aggravating ischemic brain injury. Meanwhile, excessive ROS also rapidly oxidizes endogenous nitric oxide (NO) and reduces its bioavailability, impairing vascular protection and neuroprotection. Therefore, synchronous ROS scavenging and NO generation represent a more potent and comprehensive strategy for ischemic stroke treatment (Figure 2A). Prussian blue nanoparticles have been widely verified to exhibit outstanding multi‐enzyme mimetic and ROS‐scavenging properties [30, 31]. Accordingly, we systematically evaluated the ROS‐scavenging performance, NO production kinetics, and their synergistic mechanism of RM@HPAN.

**FIGURE 2:**
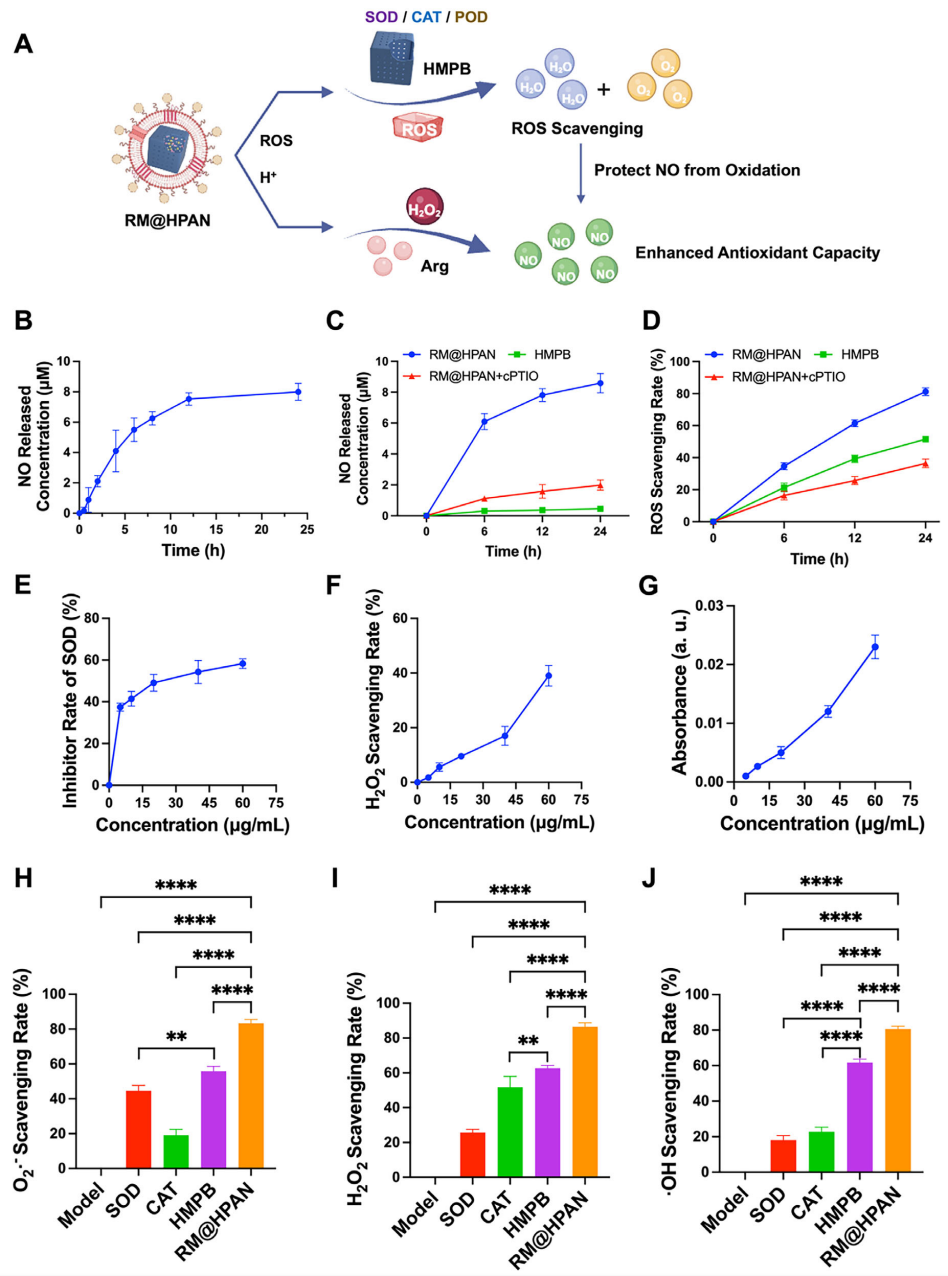
ROS‐Scavenging Capacities, NO Generation, and Synergistic Mechanism of RM@HPAN. (A) Schematic of ROS scavenging and NO generation synergistic mechanism in RM@HPAN. (B) NO production by RM@HPAN at different times. (E) The SOD catalytic activity of RM@HPAN. (F) The CAT catalytic activity of RM@HPAN. (G) The POD catalytic activity of RM@HPAN under different concentrations. (H) O_2_•^−^ scavenging activity of different samples at 200 µg/mL measured by WST‐8 method. (I) H_2_O_2_ scavenging activity of different samples at 200 µg/mL determined by CAT assay kit. (J) •OH scavenging activity of different samples at 200 µg/mL detected by TMB method. Data were expressed as mean ± SD (*n* = 3). *P < 0.05, **P < 0.01, ***P < 0.001, ****P < 0.0001, n.s. No significant. Data were analyzed by one‐way ANOVA with Tukey's post hoc test.

First, we employed the Griess method to evaluate the ability of RM@HPAN to release NO. A standard curve for NO quantification was established with excellent linearity (*y* = 0.0107*x* + 0.0421, *R* ^2^ = 0.9996). The NO release kinetics of RM@HPAN were examined under H_2_O_2_ stimulation. The results showed that NO release increased continuously in a time‐dependent manner and reached 8.0 ± 0.56 µM at 24 h (Figure 2B). Notably, synchronous ROS detection confirmed that NO production was positively correlated with ROS elimination efficiency, and the ROS‐scavenging capacity was notably weakened after NO depletion by cPTIO (NO scavenger), verifying a synergistic effect between NO generation and ROS clearance (Figure 2C,D). This synergistic pattern greatly enhances the potential of RM@HPAN for ischemic stroke therapy.

O_2_•^−^ is a primary cytotoxic ROS that can be effectively scavenged by superoxide dismutase (SOD). Using a WST‐8 based SOD activity kit, we found that the SOD‐like activity of RM@HPAN was significantly enhanced with increasing concentrations (Figure 2E). At 200 µg/mL, RM@HPAN exhibited an O_2_•^−^ scavenging rate of 83.3 ± 2.3%, which was significantly higher than those of commercial SOD, CAT, and pure HMPB nanoparticles (Figure 2H). These results confirmed that RM@HPAN possesses remarkable SOD‐mimicking activity and can efficiently eliminate O_2_•^−^.

Catalase (CAT) catalyzes the decomposition of H_2_O_2_ into H_2_O and O_2_. Based on the CAT assay kit, H_2_O_2_ consumption was determined by measuring the absorbance of the colored product at 520 nm. RM@HPAN displayed concentration‐dependent CAT‐like activity (Figure 2F). At 200 µg/mL, the H_2_O_2_ scavenging rate of RM@HPAN reached 86.5 ± 2.2%, which was markedly higher than that of commercial CAT and pure HMPB (Figure 2I), demonstrating superior CAT‐mimetic performance.

Furthermore, the peroxidase (POD)‐like activity of RM@HPAN was evaluated using TMB as the chromogenic substrate. With increasing concentrations of RM@HPAN, the absorbance of oxidized TMB gradually increased, indicating robust POD‐like activity (Figure 2G). Meanwhile, the •OH scavenging rate measured by the TMB method reached 80.6 ± 1.6% at 200 µg/mL, which was significantly stronger than commercial antioxidants and single HMPB nanozyme (Figure 2J).

In summary, RM@HPAN exerts high‐efficiency SOD‐, CAT‐, and POD‐mimetic activities to comprehensively eliminate O_2_•^−^, H_2_O_2_, and •OH. Meanwhile, it continuously generates NO under oxidative stimulation, and the two processes form a mutually reinforcing synergistic effect: ROS scavenging protects NO from oxidation, while NO further neutralizes peroxynitrite and enhances the overall antioxidant effect. By integrating multi‐ROS elimination and NO production, RM@HPAN presents powerful and synergistic antioxidant capacity, providing a highly promising nanozyme strategy for alleviating oxidative stress injury in ischemic stroke.

### 2.3 In Vitro Biological Functions and Neuroprotective Performance of RM@HPAN

To evaluate the biocompatibility of RM@HPAN, we selected PC12 cells (representing brain parenchymal neurons) and bEnd.3 cells (representing the BBB endothelium) for testing. The results of the CCK‐8 assay (Figure S5) showed that after 24 h of incubation within the concentration range of 0∼200 µg/mL, RM@HPAN did not exhibit any cytotoxicity. The viability of both cell types remained above 95% (close to 100%), indicating that this nanomaterial possesses excellent cytocompatibility and is suitable for subsequent biomedical applications.

CD47 is a glycoprotein widely expressed on the cell membrane. As a key “don't eat me” signaling molecule, it negatively regulates the phagocytic clearance process of macrophages by interacting with the SIRPα receptor on the surface of macrophages. To validate the immune evasion capacity of materials coated with red blood cell membranes, phagocytosis experiments were conducted using RAW264.7 macrophages, with unmodified HMPB/Cy5.5 serving as the control. The results showed that, compared to HPAN, the uptake of M@HPAN and RM@HPAN by macrophages was significantly reduced by 50.9% and 77.1%, respectively (Figure 3A,B). To further confirm the specificity of CD47‐mediated immune evasion, this study conducted CD47 antibody blockade experiments: after pretreating RAW264.7 cells with 5 µg/mL anti‐CD47 neutralizing antibody, the phagocytosis‐inhibiting effect of the materials was completely reversed, with macrophage uptake of M@HPAN and RM@HPAN increasing 1.9‐fold and 4.2‐fold, respectively, and the final uptake levels showing no significant difference from the HPAN group (Figure S6), thereby clarifying the immune evasion mechanism mediated by the CD47‐SIRPα pathway.

**FIGURE 3:**
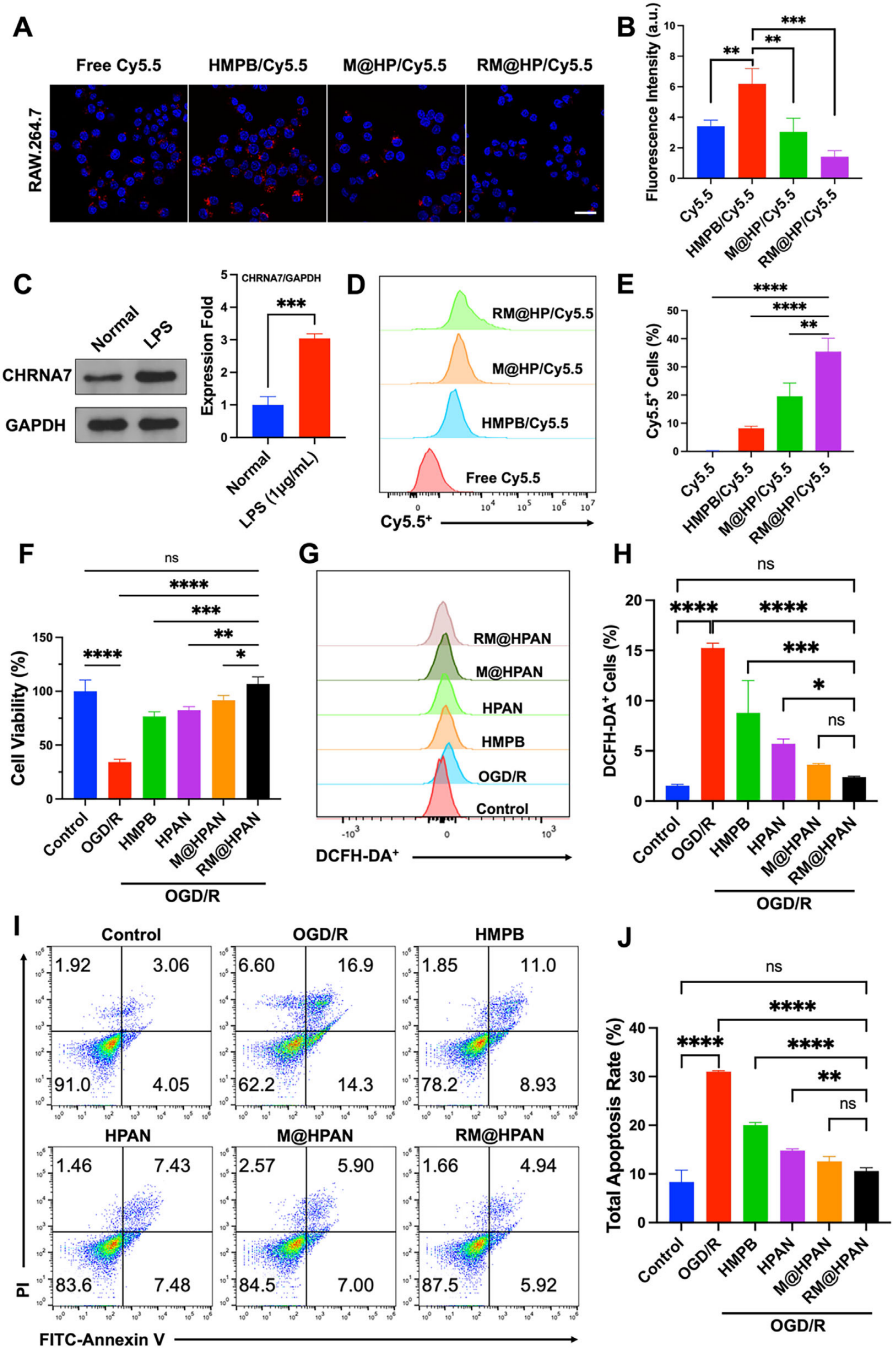
In vitro assessment of nanoparticle uptake and neuroprotection. (A) Uptake of different samples by RAW264.7 (scale bar: 20 µm) and (B) corresponding fluorescence intensity quantification. (C) CHRNA7 (α7 nAChR) expression in bEnd.3 cells under normal and LPS‐induced inflammatory conditions (1 µg/mL LPS, 12 h). (D) Flow cytometric analysis of cellular uptake of different Cy5.5‐labeled nanocarriers by LPS‐stimulated bEnd.3 cells and (E) the corresponding quantitative plots. (F) Cell viability of different nanoparticle‐treated PC12 cells in the OGD/R model. (G) Flow cytometry was used to detect the ROS scavenging efficiency of PC12 cells treated with different nanoparticles in the OGD/R model and (H) the corresponding quantitative plots. (I) Flow cytometry was used to detect the effect of different samples on the apoptosis of PC12 cells and (J) the quantitative plot of apoptosis rate. Data were expressed as mean ± SD (*n* = 3). *P < 0.05, **P < 0.01, ***P < 0.001, ****P < 0.0001, n.s. No significant. Data were analyzed by one‐way ANOVA with Tukey's post hoc test.

Meanwhile, RM@HPAN nanoparticles surface‐functionalized with the brain‐targeting peptide RVG‐29 can specifically recognize nAChRs highly expressed on bEnd.3 cells, thereby mediating active endocytosis of nanoparticles [32, 33, 34]. To verify the pathological upregulation of the RVG‐29 target receptor in the ischemic stroke microenvironment, we first examined the expression of CHRNA7 (encoding the α7 subunit of nAChR) in bEnd.3 cells under normal and lipopolysaccharide (LPS)‐induced inflammatory conditions. As shown in Figure 3C, western blot analysis confirmed that treatment with 1 µg/mL LPS significantly increased CHRNA7 expression, with a 3.0‐fold upregulation compared to the normal group, providing a pathological basis for enhanced RVG‐29‐mediated targeting. We first evaluated the cellular uptake of different nanoparticles in normal bEnd.3 cells. As shown in the results, RM@HP/Cy5.5 displayed superior internalization efficiency, with intracellular fluorescence intensity 4.5‐fold, 2.1‐fold, and 1.2‐fold higher than that of free Cy5.5, HMPB/Cy5.5, and M@HP/Cy5.5, respectively (Figure S7). Nevertheless, the fluorescence intensity in the RM@HP/Cy5.5 group was slightly higher than that in the M@HP/Cy5.5 group, but the difference was not statistically significant. To further mimic the inflammatory microenvironment of ischemic stroke in vivo, an inflammatory cellular model was established in bEnd.3 cells stimulated by 1 µg/mL LPS to investigate the uptake behavior of nanocarriers under pathological conditions. The results revealed that under inflammatory circumstances, the cellular uptake of RM@HPAN was 2.0‐fold and 4.4‐fold higher than that of M@HPAN and HMPB/Cy5.5, respectively (Figure 3D,E). Collectively, these results verify that RVG‐29 modification markedly enhances the targeting and accumulation capacity of nanoparticles toward blood–brain barrier (BBB) endothelial cells within pathological inflammatory microenvironments.

During cerebral ischemia‐reperfusion injury, excessive bursts of reactive oxygen species (ROS) degrade endogenous nitric oxide (NO) and reduce its bioavailability, thereby impairing vasodilation and neural homeostasis [35, 36, 37, 38]. Analysis using the DAF‐FM DA fluorescent probe revealed that RM@HPAN significantly increased intracellular NO levels in bEnd.3 cells via its loaded L‐arginine (Figure S8), effectively restoring NO bioavailability. Subsequently, an oxygen‐glucose deprivation/reperfusion (OGD/R) model was employed to simulate neuronal ischemia‐reperfusion injury. Neurons in the PBS control group exhibited extensive apoptotic death, confirming the successful establishment of the model; treatment with HMPB or HPAN alone demonstrated only limited neuroprotective effects, whereas RM@HPAN significantly increased PC12 cell survival to 106.8%, even exceeding that of the normal blank control group, demonstrating potent neuroprotective effects (Figure 3F). To elucidate the independent role of the L‐arginine‐mediated NO pathway, this study employed the non‐selective nitric oxide synthase inhibitor L‐NAME (1 mM) to conduct mechanism‐targeting experiments. The results showed that, compared with the OGD/R model group, RM@HPAN increased PC12 cell survival by 64.6% and reduced apoptosis by 74.6%; Following L‐NAME inhibition, these neuroprotective effects were partially reversed, with cell survival rates decreasing by 12.2% and apoptosis rates increasing by 18.7% (compared to the uninhibited RM@HPAN group, Figure S9). These results confirm that L‐arginine exerts its protective effects by mediating vasodilation through NO production and inhibiting inflammatory pathways, forming a bidirectional synergistic protective effect with the ROS‐scavenging activity of the Prussian blue core; the two represent complementary therapeutic mechanisms. Furthermore, to verify the independent pharmacological effects of NBP, RM@HPAN was compared with RM@HP (without NBP) in the OGD/R model. The results showed that RM@HPAN exhibited significantly superior protective effects: cell survival was 22% higher, and apoptosis was reduced by 39.4% (Figure S9). Based on existing literature on the mechanisms of action, NBP exerts its effects by improving cerebral blood flow and inhibiting neuronal apoptosis, which synergizes with the system's ROS scavenging and NO delivery effects to achieve multi‐pathway synergistic neuroprotection.

Furthermore, detection of intracellular ROS levels via DCFH‐DA fluorescence staining revealed a significant accumulation of ROS in the OGD/R model group; HMPB and HPAN only slightly reduced ROS levels, whereas RM@HPAN lowered intracellular ROS levels to levels comparable to those of the normal control group (Figure S10). Quantitative flow cytometry results further confirmed the superior broad‐spectrum ROS scavenging capacity of RM@HPAN (Figure 3G,H), collectively demonstrating its ability to effectively alleviate oxidative stress‐mediated cellular damage.

Finally, Annexin V‐FITC/PI double staining was used to assess neuronal apoptosis (Figure 3I,J). The results showed that the apoptosis rate in the OGD/R model group reached as high as 31%; following treatment with the materials, apoptosis levels decreased across the board. Specifically, the apoptosis rates in the RM@HPAN and M@HPAN treatment groups decreased to 10.6% and 12.6%, respectively, which was significantly better than those in the HMPB group (20.0%) and the HPAN group (14.8%). RM@HPAN demonstrated the strongest anti‐apoptotic effect, further validating its ability to alleviate OGD/R‐induced neuronal damage through multiple pathways, including BBB targeting, immune evasion, ROS scavenging, NO delivery, and synergistic effects of multi‐functional molecules.

In summary, RM@HPAN possesses excellent cellular compatibility and the ability to evade the immune system via the CD47‐mediated mononuclear phagocyte system. Leveraging the brain‐targeting and enrichment effects of RVG‐29, the broad‐spectrum ROS scavenging capacity of the Prussian blue nanozyme, the L‐arginine‐derived NO delivery pathway, and the neuroprotective pharmacological effects of NBP, as summarized in Table S2, which details the core functions and validation experiments of each component, these multiple mechanisms synergistically exert significant in vitro neuroprotective effects.

### 2.4 Dual‐Mode Brain Targeting and BBB Crossing Capacity of RM@HPAN

Based on the ability of the RVG‐29 peptide to specifically bind to nAChRs on the surface of brain microvascular endothelial cells (bEnd.3) and facilitate BBB penetration, we constructed an in vitro BBB model to evaluate the barrier‐crossing transport capacity of the nanoparticles. Specifically, the RVG‐29 peptide can recognize nAChRs highly expressed on brain microvascular endothelial cells and primarily drive the nanoplatform to traverse the intact BBB via receptor‐mediated transcytosis for active targeting. Meanwhile, local inflammation induced by ischemia‐reperfusion injury impairs BBB integrity and elevates barrier permeability, which further accelerates passive diffusion and regional accumulation of RM@HPAN within ischemic brain tissue.

In this model, bEnd.3 cells were cultured in the upper chamber, while PC12 neuronal‐like cells were cultured in the lower chamber (Figure 4A). The transendothelial electrical resistance (TEER) stably exceeded 450 Ω·cm^2^, confirming the formation of a complete and tight monolayer barrier by the bEnd.3 cells (Figure S11). Fluorescence imaging results showed that the RM@HP/Cy5.5 group exhibited the strongest fluorescence signal, with intensities 10.8, 4.5, and 3.9 times higher than those of the free Cy5.5, HMPB/Cy5.5, and M@HP/Cy5.5 groups, respectively (Figure 4B,C). This indicates that RM@HP/Cy5.5 can more effectively traverse the BBB model and be taken up by the PC12 cells in the lower chamber.

**FIGURE 4:**
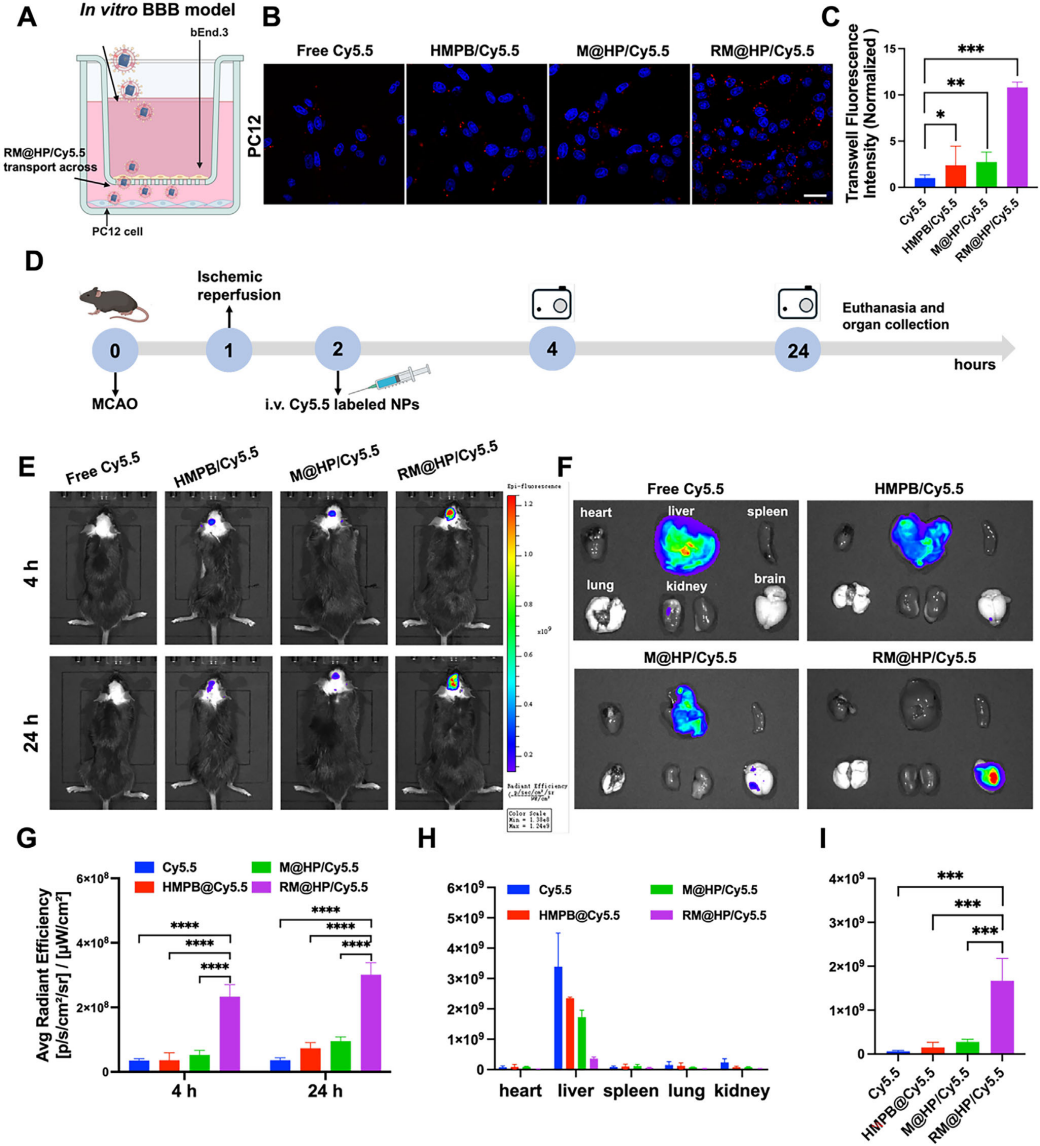
In vitro and in vivo validation of BBB penetration and brain targeting. (A) Scheme of an in vitro transwell study on co‐culture of bEnd.3 cells (up) and PC12 cells (down). (B) Uptake of free Cy5.5, HMPB/Cy5.5, M@HP/Cy5.5, and RM@HP/Cy5.5 by PC12 cells in the lower layer of BBB model (scale bar: 20 µm) and (C) the corresponding relative fluorescence quantification. (D) Schematic diagram of the in vivo experimental process. (E) In vivo fluorescence imaging and (G) corresponding quantification fluorescence of MCAO mice administrated with Cy5.5, HMPB/Cy5.5, M@HP/Cy5.5 and RM@HP/Cy5.5. (F) Ex vivo organs fluorescence imaging (all ex vivo brain tissues were uniformly positioned in the standard anatomical posture with the olfactory bulb end facing down and the brainstem end facing up) and (H, I) corresponding quantification fluorescence of the major organs of MCAO mice administrated with Cy5.5, HMPB/Cy5.5, M@HP/Cy5.5 and RM@HP/Cy5.5 at 24 h. Data were expressed as mean ± SD (*n* = 3). *P < 0.05, **P < 0.01, ***P < 0.001, ****P < 0.0001. Data were analyzed by one‐way ANOVA with Tukey's post hoc test.

To further validate its targeting capability in vivo, we utilized a MCAO mouse model. After 1 h of reperfusion, free Cy5.5, HMPB/Cy5.5, M@HP/Cy5.5, and RM@HP/Cy5.5 were administered intravenously. Whole‐body fluorescence images were captured at 4 and 24 h post‐injection using a small animal in vivo imaging system. At 24 h, the mice were sacrificed, and the brains along with major organs were harvested for ex vivo imaging (Figure 4D).

Both in vivo and ex vivo imaging results consistently demonstrated that the RM@HP/Cy5.5 group exhibited significantly superior brain‐targeting efficiency compared to all other groups. At 4 h post‐injection, although the brain signals in all groups were relatively weak, faint fluorescence was already detectable in the brain region of the RM@HP/Cy5.5 group. By 24 h, the brain fluorescence signal in this group had significantly intensified and was more localized. In contrast, the fluorescence signals of groups such as free Cy5.5 and HMPB/Cy5.5 were primarily distributed in mononuclear phagocyte system organs like the liver and spleen, with no significant enrichment observed in the brain region (Figure 4E,F). This phenomenon suggests that RM@HP/Cy5.5 possesses a time‐dependent brain‐targeting accumulation capability.

Quantitative analysis of brain fluorescence efficiency further confirmed these observations (Figure 4G). The average fluorescence efficiency of the RM@HP/Cy5.5 group at both 4 and 24 h was significantly higher than that of all other groups. Furthermore, the signal at 24 h showed an approximately 1.3‐fold increase compared to that at 4 h, indicating effective retention and continuous accumulation within the brain. Fluorescence quantification across major organs (Figure 4H) revealed that the brain signal intensity in the RM@HP/Cy5.5 group was significantly higher than that in non‐target organs such as the heart, liver, and spleen. In contrast, the brain signals in other control groups showed no significant difference compared to those in organs like the liver and spleen. Additionally, separate quantitative analysis of brain tissue fluorescence intensity (Figure 4I) clearly showed that the brain accumulation in the RM@HP/Cy5.5 group was extremely significantly higher than in all control groups, fully confirming its excellent brain‐targeting specificity. Furthermore, compared to recently reported RVG‐29 targeted nanosystems for ischemic stroke (Table S3), RM@HPAN achieved a 6.0‑fold increase in brain enrichment relative to non‑targeted controls, while reducing liver non‑targeted distribution to only 0.18‑fold of that of non‑targeted controls—markedly outperforming previously reported systems (e.g., RVG‑EVs: 1.3‑fold brain enrichment, 0.75‑fold liver distribution; RVG29‑PNPs@si‑IRF9: 2.0‑fold brain enrichment, 0.7‑fold liver distribution).

In conclusion, RM@HP/Cy5.5 demonstrates highly efficient brain‐targeting capability both in vitro and in vivo. This superior targeting performance originates from dual‐targeting effects: active transcytosis targeting driven by surface‐modified RVG‐29 peptide, passive enrichment caused by inflammation‐induced BBB leakage after ischemia‐reperfusion, as well as the long‐circulating property endowed by red blood cell membrane coating. Together, these features enable precise delivery and prolonged retention in the ischemic‐injured brain region.

### 2.5 Therapeutic Efficacy of RM@HPAN in Ischemic Stroke

To further validate the neuroprotective efficacy of RM@HPAN, we conducted systematic animal experiments using a mouse middle cerebral artery occlusion (MCAO) model. The experimental dosing regimen is illustrated in Figure 5A. In vivo T2‐weighted magnetic resonance imaging (MRI) was performed to quantitatively evaluate cerebral ischemic injury at the predetermined time point after reperfusion (Figure S12). Representative coronal brain MR images with manually delineated infarct regions are displayed in Figure S12A. Quantitative volumetric analysis revealed that the Saline group displayed substantial infarct volume. Treatment with HMPB, HPAN, and M@HPAN achieved gradual yet limited mitigation of ischemic lesions. Notably, RM@HPAN administration yielded a striking reduction in infarct volume compared with all other treatment cohorts (Figure S12B). Consistently, triphenyltetrazolium chloride (TTC) staining at 14 days post‑reperfusion (Figure 5B) showed that the saline‑treated group exhibited a large white infarct area corresponding to 29.4% of total brain volume. HMPB, HPAN, or M@HPAN treatment produced only modest infarct reductions. In contrast, RM@HPAN treatment dramatically reduced the infarct area to 3.8%, approaching the level of the sham‑operated group (Figure 5C). This superior efficacy is attributed to the prolonged circulation (erythrocyte membrane coating), active BBB targeting (RVG‑29 modification), and intrinsic ROS‑scavenging and repair‑promoting properties.

**FIGURE 5:**
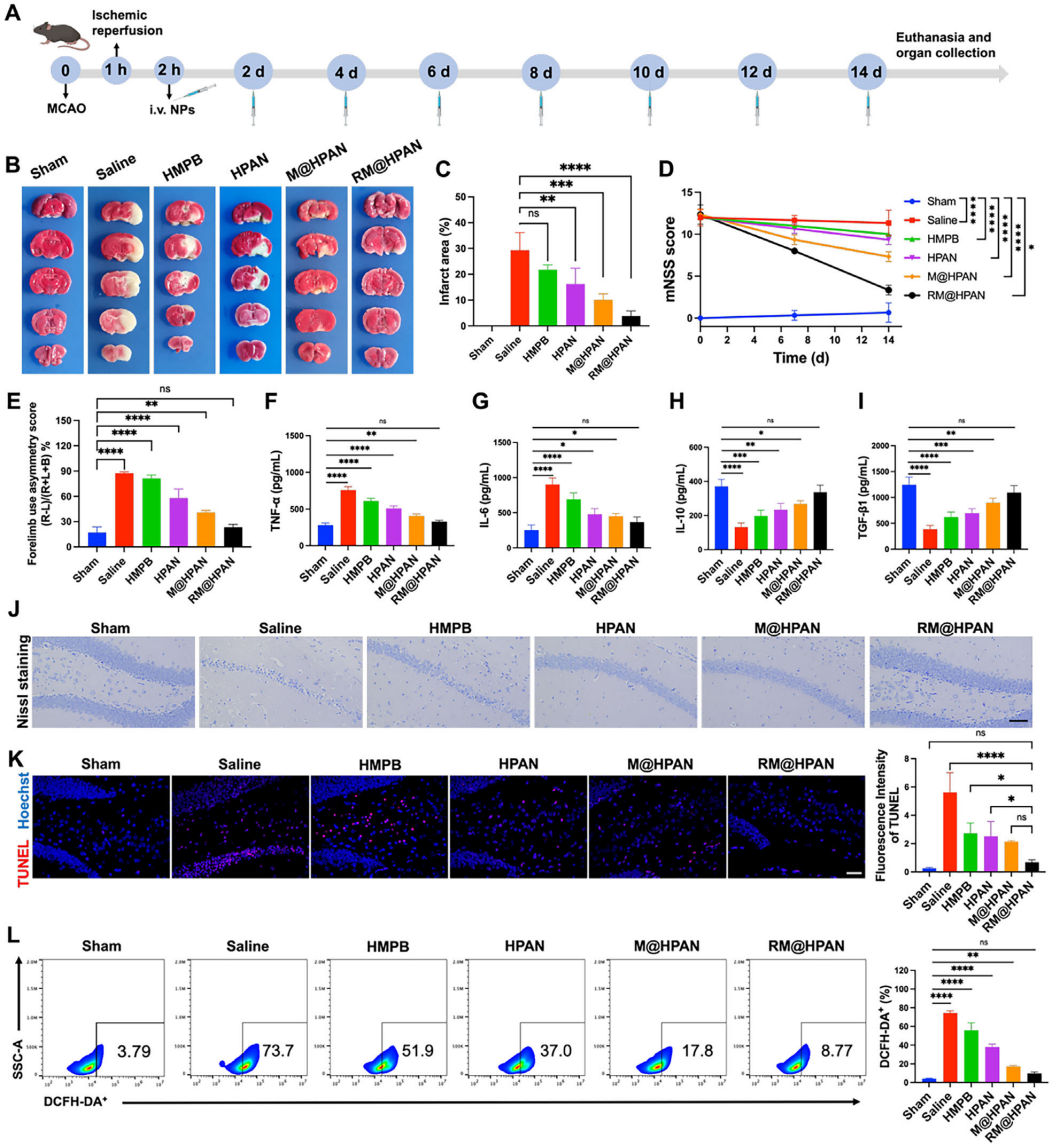
Therapeutic efficacy of RM@HPAN for ischemic stroke. (A) Schematic diagram of the in vivo experimental process. (B) Representative TTC staining images of brain slices. The red and white areas represent viable tissue and necrotic tissue, respectively, and (C) corresponding quantification of regions of cerebral infarction. (D) Neurological function evaluated using mNSS scores in different treatment groups. (E) Functional recovery evaluated using cylinder test in different treatment groups. Expression levels of inflammatory cytokines TNF‐α (F), IL‐6 (G), IL‐10 (H), and TGF‐β1 (I) in the ischemic penumbra, measured by ELISA. (J) Nissl staining imaging of brain regions in MCAO mice (scale bar: 20 µm). (K) Typical fluorescence images of TUNEL staining in the ischemic penumbra of various groups of MCAO mice (scale bar: 40 µm) and corresponding quantitative plots. (L) Flow cytometry analysis of ROS levels in the brain following different treatments, and the data were quantified. Data were expressed as mean ± SD (n = 3). *P < 0.05, **P < 0.01, ***P < 0.001, ****P < 0.0001, n.s. No significant. Data were analyzed by one‐way ANOVA with Tukey's post hoc test.

We next evaluated neurological function recovery. Modified neurological severity score (mNSS) at 0, 7, and 14 days post‑modeling (Figure 5D) showed that RM@HPAN treatment resulted in time‑dependent neurological improvement. By day 14, the mNSS score of the RM@HPAN group was not statistically different from that of the sham group, while the saline group remained severely impaired. The cylinder test (Figure 5E) revealed that the forelimb use asymmetry score in the RM@HPAN group (23.4%) was significantly lower than in the saline (87.4%), HMPB (81.2%), HPAN (58.0%), and M@HPAN (41.0%) groups, confirming effective restoration of motor coordination.

To explore the anti‑inflammatory mechanism, we measured cytokine levels by ELISA (Figure 5F–I). RM@HPAN significantly reduced pro‑inflammatory cytokines TNF‑α (from 759.7 pg/mL in the saline group to 328.0 pg/mL) and IL‑6 (from 901.8 pg/mL to 366.0 pg/mL), while elevating anti‑inflammatory cytokines IL‑10 (from 132.3 pg/mL to 335.8 pg/mL) and TGF‑β1 (from 385.6 pg/mL to 1094.6 pg/mL). Neuronal protection was further assessed by Nissl and TUNEL staining. Nissl staining (Figure 5J) showed that RM@HPAN preserved abundant Nissl bodies in the peri‑infarct region, whereas the saline group exhibited sparse and disorganized Nissl bodies. TUNEL staining (Figure 5K) revealed that RM@HPAN dramatically reduced the number of apoptotic cells, with anti‑apoptotic efficacy superior to that of HMPB or HPAN alone. Finally, DCFH‑DA staining (Figure 5L) showed that RM@HPAN treatment resulted in the lowest ROS levels, confirming potent antioxidant capacity. Additionally, NO levels in brain tissue measured by the Griess method (Figure S13) were significantly elevated in the RM@HPAN group, supporting synergistic NO‑mediated anti‑inflammatory and vasoprotective effects. Collectively, RM@HPAN exerts synergistic neuroprotective effects via multiple pathways: reducing infarct volume, improving neurological and motor function, rebalancing inflammatory cytokines, inhibiting apoptosis, and alleviating oxidative stress.

### 2.6 Validation of Key Signaling Pathways: Rap1/PI3K/Akt and Nrf2/HO‐1

To directly validate the molecular mechanisms underlying the observed therapeutic effects, we performed Western blot, ELISA, and biochemical assays for the key pathways identified by subsequent omics analyses. As illustrated in Figure 6A, RM@HPAN activated the Rap1/PI3K/Akt cascade. Western blot (Figure 6B–E) showed that compared with the saline group, RM@HPAN significantly increased the active form Rap1‑GTP and total Rap1, as well as the phosphorylation levels of PI3K (p‑PI3K) and Akt (p‑Akt). Concomitantly, RM@HPAN reduced the levels of phosphorylated NF‑κB (p‑NF‑κB), the pro‑apoptotic proteins Bax and cleaved caspase‑3 (CASP‑3), while upregulating the anti‑apoptotic protein Bcl‑2. ELISA measurements (Figure 6F–I) further confirmed that RM@HPAN reduced TNF‑α and IL‑6 and elevated IL‑10 and TGF‑β1, consistent with suppression of NF‑κB‑driven inflammatory transcription.

**FIGURE 6:**
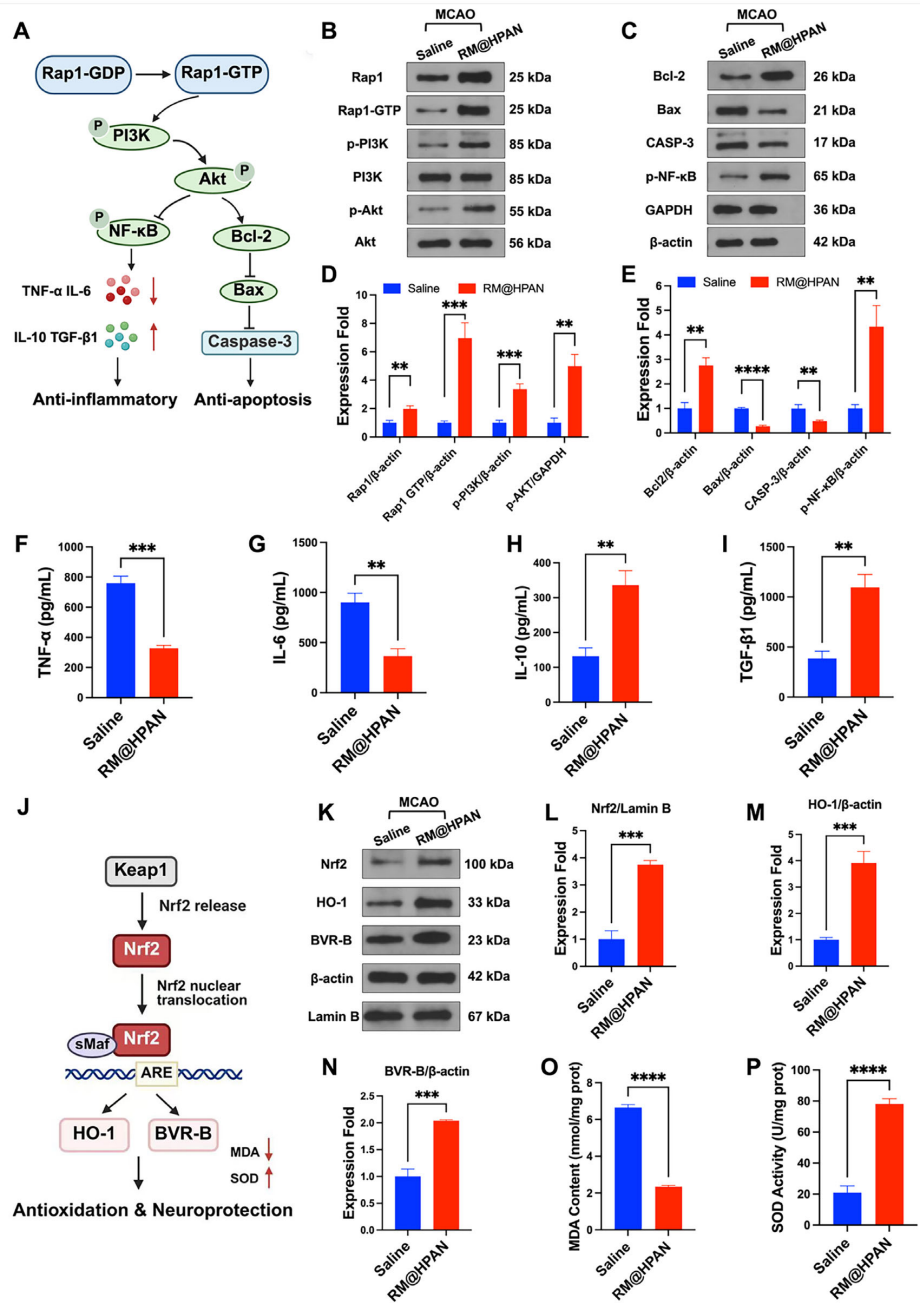
Activation of the Rap1/PI3K/Akt and Nrf2/HO‐1 signaling pathways by RM@HPAN. (A) Schematic diagram of the Rap1/PI3K/Akt‐mediated neuroprotective signaling pathway. (B,C) Western blot analysis of the expression levels of key proteins in the Rap1/PI3K/Akt pathway, including Rap1, Rap1‐GTP, p‐PI3K, PI3K, p‐Akt, Akt, Bcl‐2, Bax, Caspase‐3, and p‐NF‐κB. (D,E) Quantification of the relative expression of pathway‐related proteins (normalized to β‐actin or GAPDH). (F–I) ELISA detection of the concentrations of pro‐inflammatory cytokines (TNF‐α, IL‐6) and anti‐inflammatory cytokines (IL‐10, TGF‐β1) in brain tissue homogenates. (J) Schematic diagram of the Nrf2‐mediated antioxidant signaling pathway. (K) Western blot analysis of nuclear Nrf2, HO‐1, and BVR‐B expression in brain tissues. Lamin B and β‐actin were used as loading controls for nuclear and total proteins, respectively. (L–N) Quantification of relative protein expression. (O,P) Biochemical detection of MDA content and SOD activity in brain tissue homogenates. Data were expressed as mean ± SD (*n* = 3). *P < 0.05, **P < 0.01, ***P < 0.001, ****P < 0.0001, n.s. No significant. Data were analyzed by two‐tailed Student's t‐test.

For the antioxidant pathway (Figure 6J), Western blot analysis of nuclear fractions (Figure 6K–N) revealed that RM@HPAN promoted Nrf2 nuclear translocation (approximately 3‑fold increase), leading to upregulation of its target genes HO‑1 and BVR‑B. Biochemical assays (Figure 6O,P) showed that RM@HPAN significantly reduced malondialdehyde (MDA) content and increased superoxide dismutase (SOD) activity. These results directly link the Nrf2/HO‐1 pathway to the antioxidant function of RM@HPAN, complementing the direct ROS‑scavenging activity of the HMPB nanozyme. Together, these validations establish that RM@HPAN exerts neuroprotection through coordinated activation of the Rap1/PI3K/Akt (anti‑inflammatory/anti‑apoptotic) and Nrf2/HO‐1 (antioxidant) pathways, in addition to its direct NO‑releasing and ROS‑scavenging functions.

### 2.7 Transcriptomic Analysis and Its Causal Association With RM@HPAN Functional Modules

To establish causal links between transcriptomic changes and the specific functional modules of RM@HPAN (antioxidant, anti‑inflammatory, and immunomodulatory), we performed RNA‑seq analysis on brain tissues from saline‑ and RM@HPAN‑treated MCAO mice. As shown in Figure 7A–C, 578 differentially expressed genes (DEGs) were identified (|log_2_FC|>1, *p* < 0.05), including 226 upregulated and 352 downregulated genes. The heatmap and volcano plot clearly separate the two groups, indicating extensive transcriptional reprogramming by RM@HPAN.

**FIGURE 7:**
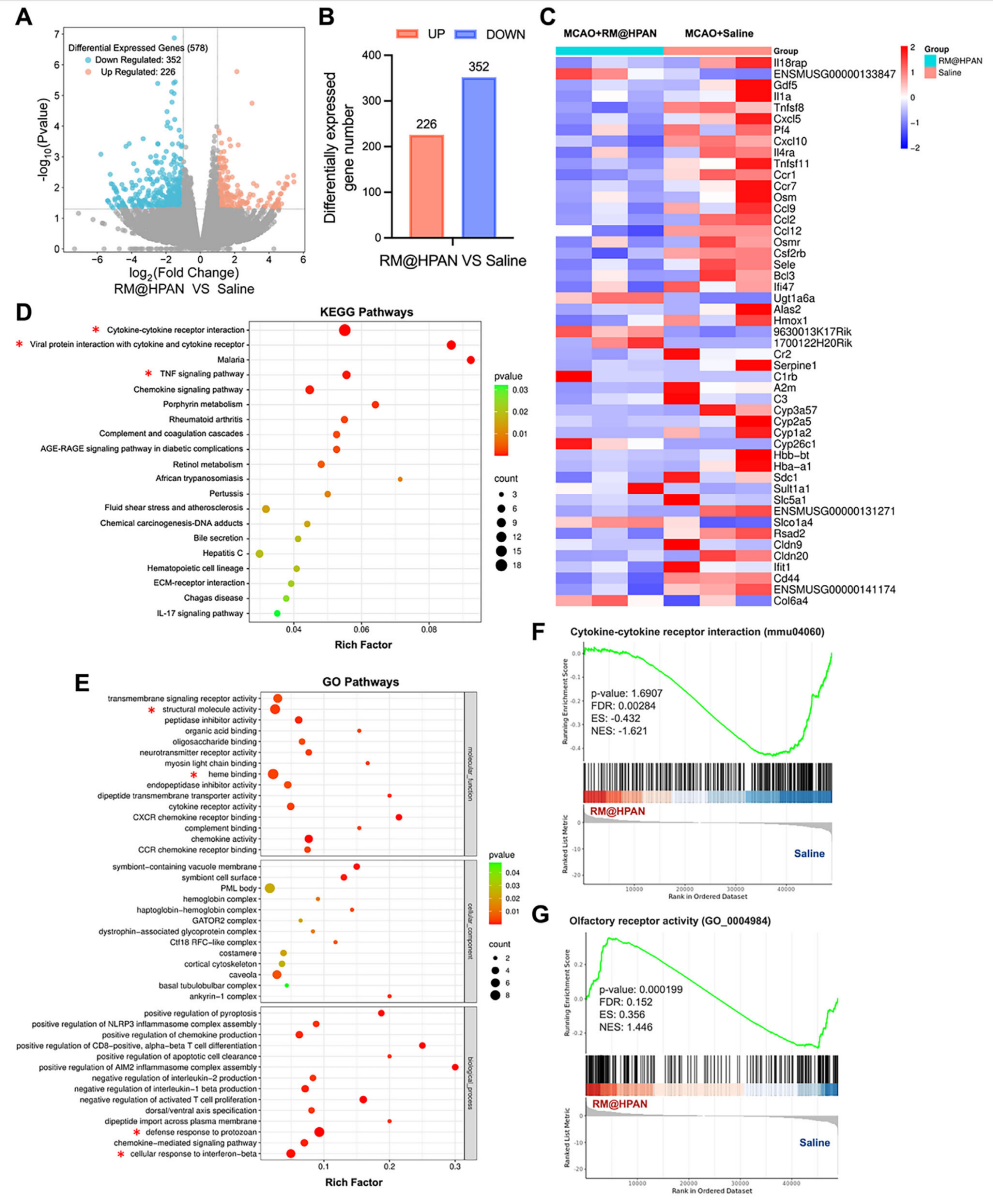
Transcriptome analysis revealed RM@HPAN‐induced gene expression changes and pathway enrichment. (A) Volcano plot of significantly upregulated (red) and downregulated (blue) genes (|log_2_FC|>1, p < 0.05). (B) Bar chart showing the number of up‐ and down‐regulated DEGs. (C) Heatmap of DEGs between the control group (saline) and RM@HPAN‐treated group. (D) KEGG pathway enrichment analysis of DEGs. (E) GO enrichment analysis of DEGs. (F) GSEA plot for representative genes in the selected pathway: Cytokine‐cytokine receptor interactions. (G) Representative GSEA plot for the selected pathway: Olfactory receptor activity.

Kyoto Encyclopedia of Genes and Genomes (KEGG) pathway enrichment analysis (Figure 7D) showed that the DEGs were primarily enriched in “cytokine‐‑cytokine receptor interaction,” “TNF signaling pathway,” “chemokine signaling pathway,” and “viral protein interaction with cytokine and cytokine receptor” [39, 40]. These pathways are directly linked to RM@HPAN's NO‑releasing function (derived from L‑arginine, Figure 2B‐D). NO is known to inhibit NF‑κB nuclear translocation and suppress the transcription of pro‑inflammatory cytokines. Consistent with this, our transcriptomic data showed significant downregulation of TNF‐α and IL‐6 and upregulation of anti‑inflammatory genes IL‐10 and TGF‐β1. Additionally, the enrichment of “fluid shear stress and atherosclerosis” reflects the erythrocyte membrane‑mediated immune evasion (CD47, Figure S6), which reduces macrophage phagocytosis and modulates vascular inflammation.

Gene ontology (GO) pathway enrichment analysis (Figure 7E) revealed enrichment of terms such as “heme binding,” “cellular response to interferon‑beta,” and “defense response to protozoan” [41, 42]. Heme binding is directly relevant to the Nrf2/HO‐1 antioxidant pathway validated above as HO‑1 is a heme oxygenase. The interferon‑related terms reflect the immunomodulatory effects of NO and the reduction of oxidative stress, which can dampen type I interferon responses in ischemia.

Gene Set Enrichment Analysis (GSEA) (Figure 7F) demonstrated significant negative enrichment of the “cytokine‑‐cytokine receptor interaction” pathway (NES = −1.621). This indicates that RM@HPAN suppresses the upstream signaling of this pathway, reducing activation of NF‑κB and downstream inflammatory gene expression. This is a direct causal consequence of the NO‑mediated immunomodulation and nanozyme‑mediated ROS scavenging, both of which inhibit the inflammatory cascade. The “olfactory receptor activity” pathway was significantly positively enriched (NES = 1.446, Figure 7G). Although unexpected, olfactory receptors are expressed in the brain and have been implicated in neurogenesis and neuronal repair. This positive enrichment may represent a repair‑related transcriptional response induced by RM@HPAN, possibly via activation of the Rap1/PI3K/Akt pathway. We have integrated the transcriptomic data into the following causal framework: RM@HPAN → NO release + ROS scavenging → suppression of NF‑κB → downregulation of cytokine‑cytokine receptor interaction and TNF signaling → reduced neuroinflammation.

### 2.8 Proteomic Analysis and Its Integration With Transcriptomic Data

Proteomic analysis was performed to complement transcriptomic findings and to resolve the apparent discrepancy between the two datasets. The analysis identified 150 DEPs (84 upregulated, 66 downregulated; |log_2_FC|>1, *p* < 0.05) in RM@HPAN‑treated mice compared with saline controls (Figure 8A,B). While transcriptomic enrichment captured upstream inflammatory signaling, proteomic enrichment (Figure 8C,D) identified downstream metabolic and functional execution pathways, including “amide metabolic process,” “generation of precursor metabolites and energy,” “Rap1 signaling pathway,” “platelet activation,” and “ECM‑receptor interaction” [43]. This discrepancy reflects temporal hierarchy: transcriptome (early) shuts down inflammatory gene expression; proteome (late) executes metabolic and repair programs.

**FIGURE 8:**
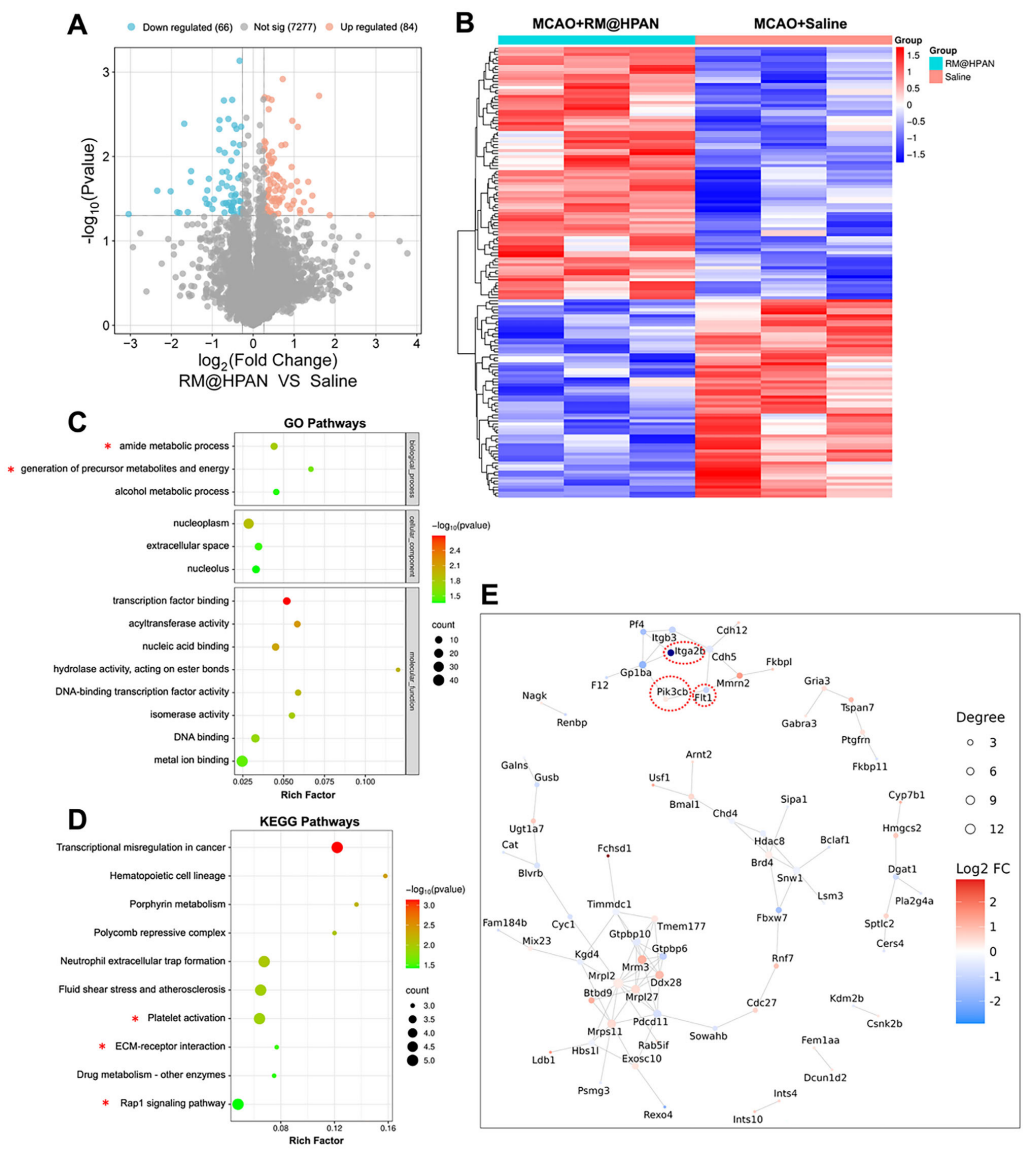
Proteomic analysis revealed RM@HPAN‐induced protein expression changes, pathway enrichment, and interaction networks. (A) Volcano plot of significantly upregulated (red) and downregulated (blue) proteins (|log_2_FC|>1, *p* < 0.05). (B) Heatmap of DEPs between the control group (saline) and RM@HPAN‐treated group. (C) GO enrichment analysis of DEPs. (D) KEGG pathway enrichment analysis of DEPs. (E) Diagram of the protein interaction network.

Notably, in the proteomic KEGG enrichment analysis (Figure 8D), although the Rap1 signaling pathway showed moderate enrichment with the lowest Rich Factor, it acts as the central functional signaling hub mediating RM@HPAN‑triggered neuroprotection. This apparent inconsistency between enrichment strength and biological importance is not uncommon as key regulatory pathways often involve only a limited number of core proteins that are tightly controlled at the post‑translational level. Consistent with this notion, our targeted Western blot validation (Figure 6B–E) confirmed that RM@HPAN significantly upregulates Rap1‑GTP, p‑PI3K, and p‑Akt, while downregulating p‑NF‑κB, Bax, and Caspase‑3, providing direct evidence for the functional centrality of the Rap1 pathway despite its moderate enrichment score.

The “Platelet activation” and “Hematopoietic cell lineage” pathways are linked to erythrocyte membrane coating (CD47) and reduced platelet aggregation [44, 45], as supported by the downregulation of the hub protein Itga2b in the PPI network (Figure 8E) [46]. The PPI network also identified other hubs such as Pik3cb and Flt1 [47, 48], representing potential therapeutic targets. We established a unified model: transcriptomic suppression of inflammatory genes → proteomic activation of Rap1/PI3K/Akt and Nrf2/HO‐1 pathways → reduced apoptosis, oxidative stress, and platelet activation → improved neurological outcome.

### 2.9 Safety Evaluation of RM@Hpan

First, the hemocompatibility of RM@HPAN was assessed using an in vitro hemolysis assay. As shown in Figure S14, the positive control (double‐distilled water, ddH_2_O) caused significant hemolysis, while the negative control (PBS) induced almost no hemolysis. Within the concentration range of 0.1∼1.8 mg/mL, the hemolysis rates of RM@HPAN were all below the 5% safety threshold, with no significant differences observed among the different concentration groups. This indicates that the nanoparticles possess good hemocompatibility. Subsequently, a preliminary in vivo safety evaluation was conducted in healthy mice. The mice received eight injections of RM@HPAN over 14 days. Body weight was monitored throughout the study, and blood along with major organs were collected after the final administration for analysis. Biochemical analysis results showed that RM@HPAN treatment did not cause liver or kidney function damage (Figure S15). Furthermore, body weight gain trends and organ indices (heart, liver, spleen, lungs, and kidneys) showed no significant differences compared to the control group (Figure S16). Histopathological examination also revealed no apparent pathological lesions or inflammatory infiltration in the major organs (Figure S17). The results from these systematic in vitro and in vivo safety experiments demonstrate that RM@HPAN exhibits good biocompatibility and safety, laying a necessary foundation for its potential biomedical applications.

## 3 Conclusion

In this study, we developed a biomimetic hybrid nanoplatform (RM@HPAN) by coating hollow mesoporous Prussian blue nanoparticles (co‐loaded with L‑arginine and butylphthalide) with red blood cell membrane and modifying with the brain‑targeting peptide RVG‑29. RM@HPAN demonstrated potent therapeutic efficacy against ischemic stroke by integrating multi‑enzyme mimetic antioxidant activity, sustained nitric oxide release, active BBB targeting, and immune evasion. Mechanistically, RM@HPAN improved neurological outcomes by alleviating oxidative stress, modulating neuroinflammation, and inhibiting neuronal apoptosis. In vitro and in vivo experiments consistently showed that RM@HPAN reduced cerebral infarct volume (from 29.4% to 3.8%), promoted motor function recovery, rebalanced inflammatory cytokines (downregulating TNF‑α and IL‑6, upregulating IL‑10 and TGF‑β1), and decreased ROS levels in brain tissue. These results highlight the advantage of combining biomimetic membrane coating with functional nanocarriers for precision therapy of cerebral ischemia.

One of the key mechanistic findings is that RM@HPAN activated the Rap1/PI3K/Akt and Nrf2/HO‑1 signaling pathways, which are central regulators of cellular anti‑inflammatory, anti‑apoptotic, and antioxidant responses. This activation contributes to the observed reduction in oxidative damage, suppression of NF‑κB‑driven inflammation, and enhancement of neuronal survival. Nevertheless, it is worth noting that other signaling pathways may also be involved in the protective effects of RM@HPAN. For example, the MAPK and chemokine signaling pathways are well‑known regulators of inflammatory responses during ischemic injury. Although these pathways were not directly investigated in the present study, it is plausible that RM@HPAN exerts broader modulatory effects through them. Future transcriptomic or proteomic analyses with more detailed validation will be valuable to delineate the involvement of additional signaling cascades, thereby providing a more comprehensive mechanistic understanding.

Despite the promising therapeutic benefits observed, several challenges remain to be addressed before clinical translation. Large‑scale and reproducible production of RM@HPAN requires further optimization, and the long‑term safety and pharmacokinetics in large animal models need to be evaluated. Additionally, donor‑related heterogeneity of red blood cell membranes may affect batch consistency. Addressing these issues will be critical for moving RM@HPAN closer to clinical application. Beyond ischemic stroke, the versatile design of RM@HPAN holds promise for other central nervous system disorders that share pathological features of oxidative stress and neuroinflammation, such as traumatic brain injury and Alzheimer's disease. Exploring these broader applications could significantly enhance the academic scope and translational relevance of this work.

Taken together, our findings demonstrate that RM@HPAN represents a novel and multifunctional therapeutic strategy for ischemic stroke. By integrating biocompatibility, BBB penetrability, multimodal antioxidant, anti‑inflammatory, and anti‑apoptotic effects, RM@HPAN provides a strong conceptual and technical foundation for the development of next‑generation nanomedicines for CNS diseases.

## 4 Experimental Section

### 4.1 Materials

Potassium ferricyanide (K_3_[Fe(CN)_6_]), polyvinylpyrrolidone (PVP) K29‐32, and 3,3’,5,5’‐tetramethylbenzidine (TMB) were supplied by Shanghai Aladdin Biochemical Technology Co., Ltd. Dl‐3‐n‐butylphthalide (NBP), L‐arginine (Arg), and Cy5.5 were purchased from MedChemexpress. DSPE‐PEG_2000_‐RVG29 (YTIWMPENPRPGTPCDIFTNSRGKRASNG) was purchased from Guangzhou Weihua Biotechnology Co., Ltd. The WST‐8 Total Superoxide Dismutase Assay Kit, Catalase Assay Kit, NO Content Detection Kit, BCA protein Assay Kit, and Reactive Oxygen Species Detection Kit were purchased from Shanghai Biyuntian Biotechnology Co., Ltd. The CCK‐8 was purchased from Biosharp Biotechnology Co., Ltd. The Annexin V‐FITC/PI Apoptosis Detection Kit was purchased from Jiangsu Kaiji Biotechnology Co., Ltd. Histological staining reagents: 2,3,5‐Triphenyltetrazolium chloride (TTC) was purchased from Wuhan Servicebio Technology Co., Ltd. The Nissl staining kit was purchased from Qisai Biotechnology (Wuhan) Co., Ltd. TUNEL BrightRed Apoptosis Detection Kit was purchased from Nanjing Vazyme Biotechnology Co., Ltd. DAPI was purchased from Shanghai Biyuntian Biotechnology Co., Ltd. Western blot reagents: Phosphatase inhibitors, PMSF, and RIPA lysis buffer were purchased from Wuhan Servicebio Technology Co., Ltd. The PVDF membranes were purchased from Millipore Corporation. Primary antibodies (see below for details) were purchased from various manufacturers, and HRP‐conjugated secondary antibodies were obtained from Shanghai Biyuntian Biotechnology Co., Ltd. The ECL substrate solution was purchased from Wuhan Servicebio Technology Co., Ltd. ELISA kits: Mouse TNF‐α, IL‐6, IL‐10, and TGF‐β1 ELISA kits were purchased from Wuhan Fine Biotechnology Co., Ltd.

### 4.2 Cell Culture

bEnd.3 cell was cultured in DMEM containing 10% fetal bovine serum (FBS) and 1% penicillin/streptomycin at 37°C in a 5% CO_2_ incubator. PC12 cells were cultured in 1640 containing 10% FBS and 1% penicillin/streptomycin at 37°C in a 5% CO_2_ incubator. bEnd.3 and PC12 cells were obtained from Qisai Biotechnology (Wuhan) Co., LTD. (Wuhan). Cell culture reagents: DMEM (for bEnd.3), RPMI‐1640 (for PC12), FBS, penicillin/streptomycin, trypsin‐EDTA, and sugar‐free DMEM (for OGD/R) were purchased from Qisai Biotechnology (Wuhan) Co., Ltd.

### 4.3 Experimental Animals and Ethics

Female C57BL/6 mice (6–8 weeks old) were housed at the Hubei Provincial Center for Disease Control and Prevention. All animal procedures adhered to ethical guidelines for animal welfare and protection, as reviewed and approved by the Hubei Provincial Center for Disease Control and Prevention Committee for the Management and Use of Laboratory Animals (Ethics Approval No. 202510145).

### 4.4 Preparation of Hollow Mesoporous Prussian Blue Nanoparticles (HMPB)

HMPB was synthesized via a hydrothermal method. First, K_3_[Fe(CN)_6_] (132 mg) and PVP (3 g) were dissolved in 40 mL of 0.01 M hydrochloric acid solution and stirred at room temperature for 1 h until the solution turned yellow and transparent. The solution was then transferred to a three‐neck flask and heated in an 80°C water bath for 24 h. PB was obtained by frequent centrifugation and washed multiple times with deionized water and ethanol. The precipitate was dried under vacuum. Next, PB (20 mg) and PVP (100 mg) were mixed in 20 mL of hydrochloric acid (1 M) solution. After stirring for one hour, the solution was transferred to a reactor with a PTFE‐lined inner wall and heated at 140°C for 4 h. During this process, the PVP layer shielded the shell from the corrosive action of acidic H^+^ ions, thereby forming a mesoporous structure.

### 4.5 Preparation of HMPB/Arg‐NBP (HPAN)

Dissolve 10 mg HMPB and 10 mg L‐arginine (Arg) in 10 mL deionized water. Add butylphthalide (NBP, 1 mg) dissolved in ethanol dropwise to the HMPB solution. Stir the mixture in the dark for 12 h. After the reaction, centrifuge at 12,000 rpm for 10 min and wash the precipitate three times with deionized water.

### 4.6 Preparation of Red Blood Cell Membrane‐Encapsulated HPAN (M@HPAN)

Red blood cell membranes (RCM) were extracted using the hypotonic centrifugation method. Collect whole blood from C57BL/6 mice into heparin sodium centrifuge tubes. Centrifuge at 2500 rpm for 5 min in a low‐speed centrifuge at 4°C. Remove the supernatant plasma layer, retaining the dark red packed red blood cell pellet. Wash the red blood cells in the lower layer three times with 1 × PBS (1:3), then obtain the packed red blood cells by low‐speed centrifugation. Place the red blood cells in 0.25 × PBS (1:40) and perform hypotonic treatment on ice for 40∼60 min. Centrifuge at 13,000 rpm for 15 min in a high‐speed centrifuge at 4°C. Remove the lower layer of blood and wash multiple times with 0.25 × PBS until the supernatant is colorless. The resulting pink erythrocyte membrane is resuspended in 1 × PBS solution for storage at 4°C. Mix the obtained RCM with HPAN solution, sonicate in a water bath for 10 min, and repeatedly extrude approximately 10 times using a liposome extruder (Avestin LF1, Canada) with polycarbonate membranes of 400 nm and 200 nm pore sizes to obtain the biomimetic nanoparticle M@HPAN solution, which is stored at 4°C.

### 4.7 Modification of RVG‐29 Peptide

First, add 5 mg of DSPE‐PEG_2000_‐RVG29 powder to M@HPAN solution (0.5 mg/mL, 2 mL) and incubate overnight at 4°C. Then, dialyze the resulting solution in deionized water using a dialysis bag (MWCO 10 kDa) for 12 h. This yields the RM@HPAN solution.

### 4.8 Cy5.5 Labeling of Nanoparticles

To prepare Cy5.5‑labeled nanoparticles (HMPB/Cy5.5, M@HP/Cy5.5, and RM@HP/Cy5.5), the same procedure for HPAN was followed, except that L‑arginine and NBP were replaced with Cy5.5. Briefly, 10 mg of HMPB was dispersed in 10 mL deionized water, and 1 mg of Cy5.5 (dissolved in ethanol) was added dropwise. The mixture was stirred in the dark for 12 h at room temperature. The resulting Cy5.5‑loaded HMPB (HMPB/Cy5.5) was collected by centrifugation (12,000 rpm, 10 min) and washed three times with deionized water. Subsequent coating with red blood cell membrane (to obtain M@HP/Cy5.5) and RVG‑29 modification (to obtain RM@HP/Cy5.5) were performed following the same protocols as described for M@HPAN and RM@HPAN, respectively.

### 4.9 Determination of Size, Zeta Potential, and Colloidal Stability of Nanoparticles

The hydrodynamic diameter, polydispersity index (PDI), and zeta potential of nanoparticles were measured using a Malvern Zetasizer Nano ZS90 at 25°C. For colloidal stability assessment, RM@HPAN nanoparticles were dispersed in 1 × PBS (pH 7.4) at a concentration of 0.5 mg/mL and stored at 4°C. The particle size and PDI were measured at 0, 1, 3, 5 and 7 days.

### 4.10 Morphological Observation of Nanoparticles

10 µL of nanoparticle suspension (0.1 mg/mL) was dropped onto a carbon‐coated copper grid and air‐dried at room temperature. The morphology of nanoparticles was observed using a JEM‐F200 TEM at an accelerating voltage of 200 kV. Elemental mapping was performed using an energy‐dispersive x‐ray spectroscopy (EDS) detector attached to the TEM.

### 4.11 Nitrogen Adsorption–Desorption Experiments

To demonstrate that the synthesized HMPB exhibits a mesoporous surface with a hollow structure at its core, 100 mg of HMPB solid powder was obtained via freeze‐drying. Following drying at 700°C, surface pore size distribution testing was conducted using a fully automated specific surface area and pore size analyzer (Micromeritics ASAP 2460, USA).

### 4.12 XRD Characterization

10 mg each of HMPB and HPAN solid powders were obtained via freeze‐drying. Wide‐angle testing was performed on the HMPB sample using a BRUKER D8 Advance (Germany), yielding specific x‐ray diffraction peaks.

### 4.13 FTIR Characterization

Solid powders of HMPB and HPAN (5 mg each) were placed in the sample cell of an FTIR spectrophotometer (Thermo Fisher Scientific, USA). The spectra were recorded over a wavenumber range of 499 cm^−1^ to 4000 cm^−1^ to obtain the characteristic infrared absorption peaks of HMPB.

### 4.14 UV–Vis Characterization

Solutions of HMPB, NBP, Arg, and HPAN (0.05 mg/mL, 2 mL each) were sequentially added to the sample cell of a UV‐Vis spectrophotometer (Shimadzu UV‐1780, Japan). The absorption spectra were recorded over a wavelength range of 200 nm to 1000 nm to identify the characteristic UV absorption peaks of each component.

### 4.15 Determination of Drug Loading Capacity and Encapsulation Efficiency

Standard curves for NBP and Arg were established using a UV‐‑vis spectrophotometer (Shimadzu UV‑1780) at their characteristic wavelengths (NBP: 274 nm; Arg: 210 nm). The drug content in HPAN was then measured. Drug loading capacity (DL) and encapsulation efficiency (EE) were calculated according to the following formulas: DL (%) = mass of drug in nanoparticles / mass of nanoparticles × 100%, EE (%) = mass of drug in nanoparticles / mass of drug fed initially × 100%.

### 4.16 Membrane Protein Characterization

RCM and RM@HPAN were lysed in RIPA buffer containing 1% protease inhibitor on ice for 1 h. After centrifugation (13,000 rpm, 10 min, 4 °C), the supernatant was discarded, and protein content was determined by BCA assay. Equal amounts of protein were subjected to SDS‑PAGE and transferred to PVDF membranes. The expression of CD47 and Band 3 was detected by Western blot.

### 4.17 In Vitro Drug Release Testing

To investigate the pH‑dependent release profiles of NBP and Arg, samples were divided into four groups: (1) HPAN (pH 5.0), (2) HPAN (pH 7.4), (3) RM@HPAN (pH 5.0), and (4) RM@HPAN (pH 7.4). Briefly, 1 mL of HPAN solution (2 mg/mL) sealed in a dialysis bag (MWCO 10 kDa) was immersed in 20 mL of PBS buffer (pH 7.4 or pH 5.0). The same procedure was applied to RM@HPAN solution (2 mg/mL). At each time interval (every 12 h), 2 mL of dialysate was collected and replaced with an equal volume of fresh PBS. The released amounts of Arg and NBP were measured using a UV‐‑vis spectrophotometer (Shimadzu UV‑1780) at their characteristic wavelengths (Arg: 210 nm; NBP: 274 nm).

### 4.18 NO Release Behavior

L‑Arginine (Arg) loaded in RM@HPAN can generate nitric oxide (NO) upon reaction with hydrogen peroxide (H_2_O_2_). To evaluate this, RM@HPAN nanoparticles were diluted to 200 µg/mL in appropriate buffer, and 200 µL of this suspension was incubated with 10 mM H_2_O_2_. The NO release kinetics were monitored over time, and the NO content was determined at each time point using a NO detection kit (Griess method).

### 4.19 Reactive Oxygen Species Scavenging Performance

The multi‐enzyme mimetic activities of RM@HPAN were evaluated at various concentrations (0, 5, 10, 20, 40, and 60 µg/mL) using the following methods. SOD‐like activity: Superoxide dismutase (SOD)‐mimetic activity was measured using the WST‐8 Total Superoxide Dismutase Assay Kit. Briefly, 20 µL of RM@HPAN at indicated concentrations was mixed with 200 µL working solution and 20 µL xanthine oxidase solution. After 30 min of incubation, absorbance was measured at 450 nm to determine SOD catalytic activity. CAT‐like activity: Catalase (CAT)‐mimetic activity was assessed using a CAT assay kit. RM@HPAN catalyzes the decomposition of H_2_O_2_ into H_2_O and O_2_. Residual H_2_O_2_ reacts with a colorimetric substrate to produce a red product (absorption maximum at 520 nm). The absorbance at 520 nm is inversely correlated with CAT activity, which was calculated for each concentration. POD‐like activity: Peroxidase (POD)‐mimetic activity was measured at 650 nm using TMB as the chromogenic substrate in an HAc‐NaAc buffer solution containing H_2_O_2_. RM@HPAN at various concentrations was added, and the absorbance increase was recorded.

### 4.20 Cytotoxicity Assay

The cytotoxicity of RM@HPAN to PC12 cells and bEnd.3 cells was detected by CCK8 assay. Two cell lines were seeded into a 96‐well plate at 5,000 cells per well using 200 µL of medium. Subsequently, add RM@HPAN at concentrations of 0, 10, 20, 50, 100, and 200 µg/mL. After incubation for 24 h, add CCK8 reagent and continue incubation for 2 h. Measure absorbance at 450 nm and calculate cell survival rate.

### 4.21 Cell Uptake Assay

Log‐phase bEnd.3 cells (1 × 10^4^ cells) were seeded onto 12‐well plate coverslips. After 24 h of culture, cells were stimulated with 1 µg/mL LPS diluted in DMEM for 12 h. Subsequently, cells were treated with Cy5.5 (5 µg/mL), or with Cy5.5‐labeled nanoparticles (HMPB/Cy5.5, M@HP/Cy5.5, RM@HP/Cy5.5) at a nanoparticle concentration of 50 µg/mL, in which the Cy5.5 loading content was 10% (w/w), ensuring equal Cy5.5 fluorescence among all treatment groups. After 4 h of incubation, cells were washed three times with PBS, stained with Hoechst for nuclei, and observed under a confocal microscope. Cellular Cy5.5 fluorescence was also quantified by flow cytometry.

### 4.22 Assessment of Immune Evasion Capacity

The immune evasion capacity of RM@HPAN was evaluated using a macrophage phagocytosis assay. Log‐phase RAW264.7 cells (1 × 10^4^ cells) were seeded onto 12‐well glass slides and cultured for approximately 24 h. Cells were then stimulated with 1 µg/mL LPS in DMEM for 12 h, followed by 4 h of incubation with free Cy5.5 (5 µg/mL) or Cy5.5‐labeled nanoparticles (HMPB/Cy5.5, M@HP/Cy5.5, or RM@HP/Cy5.5) at a nanoparticle concentration of 50 µg/mL (with Cy5.5 loading of 10% w/w, ensuring equal Cy5.5 fluorescence across groups). Subsequently, cellular uptake was visualized using confocal microscopy. For CD47 blockade experiments, RAW264.7 cells were pretreated with 5 µg/mL anti‐CD47 neutralizing antibody before the addition of the nanoparticles, followed by the same incubation and imaging procedures as described above.

### 4.23 Neuroprotective Effects of Nanoparticles

A PC12 cell model simulating cerebral ischemia‐reperfusion injury was established using an in vitro oxygen‐glucose deprivation/recovery (OGD/R) approach. PC12 cells in logarithmic growth phase were seeded at a density of 1 × 10^5^ cells/mL in a 96‐well plate and cultured for 24 h. The original medium was replaced with 1640 sugar‐free medium, and cells underwent 6‐hour OGD treatment under 5% CO_2_, 1% O_2_, and 94% N_2_ conditions. Following OGD, cells were reoxygenated for 12 h under normoxic conditions in high‐glucose RPMI‐1640 medium supplemented with HMPB, HPAN, M@HPAN, or RM@HPAN at a concentration of 200 µg/mL (based on nanoparticle mass). Cell viability was assessed using the CCK‐8 assay.

### 4.24 Detection of ROS Levels in Cells

ROS levels in PC12 cells subjected to OGD/R and different nanoparticle treatments were measured using the DCFH‐DA fluorescent probe. After OGD/R and subsequent 12 h treatment with various nanoparticles (HMPB, HPAN, M@HPAN, RM@HPAN) in six‑well plates (at a density of 5 × 10^5^ cells/well), the culture medium was removed. Cells were then incubated with 10 µM DCFH‑DA (diluted in serum‑free medium, 1 mL/well) for 20 min at 37°C in the dark. DCFH‑DA is taken up by cells and cleaved by intracellular esterases to non‑fluorescent DCFH, which is then oxidized by ROS to fluorescent DCF. DCF fluorescence (excitation/emission: 488/525 nm) was observed under an inverted fluorescence microscope and quantified using ImageJ software. In parallel, cells were harvested by trypsinization, and DCF fluorescence was also analyzed by flow cytometry (CytoFLEX).

### 4.25 Detection of Apoptosis

The anti‑apoptotic effect of RM@HPAN was evaluated using an Annexin V‑FITC/PI apoptosis detection kit. PC12 cells after OGD/R and subsequent 12 h treatment with different nanoparticles (HMPB, HPAN, M@HPAN, RM@HPAN) in six‑well plates were harvested by trypsinization, washed with PBS, and resuspended in 1× binding buffer at a density of 1 × 10^6^ cells/mL. An aliquot of 100 µL of the cell suspension (containing 1 × 10^5^ cells) was transferred to a flow cytometry tube. Cells were then stained with 5 µL of Annexin V‑FITC for 10 min at room temperature in the dark, followed by 5 µL of propidium iodide (PI) for 5 min on ice in the dark. After staining, 400 µL of 1× binding buffer was added to each tube. Apoptosis was analyzed immediately using a flow cytometer (excitation: 488 nm; emission: 525 nm for FITC, 560 nm for PI). Data were processed using FlowJo software to quantify the percentages of early apoptotic (Annexin V^+^/PI^−^), late apoptotic (Annexin V^+^/PI^+^), and necrotic (Annexin V^−^/PI^+^) cells.

### 4.26 In Vitro BBB Permeability Study

The Transwell assay was employed to evaluate the blood‑brain barrier (BBB) crossing ability of RM@HPAN. Briefly, bEnd.3 cells were seeded at 5 × 10^4^ cells/well in the upper chamber of a 24‑well Transwell plate (pore size 1 µm) and cultured for 7 days to form a tight monolayer. The medium was changed every two days, and the transendothelial electrical resistance (TEER) was measured; only inserts with TEER values exceeding 450 Ω·cm^2^ were used for subsequent experiments. After TEER confirmation, bEnd.3 cells were stimulated with 1 µg/mL LPS for 12 h to mimic the inflammatory condition. Then, free Cy5.5 (5 µg/mL) or Cy5.5‑labeled nanoparticles (HMPB/Cy5.5, M@HP/Cy5.5, or RM@HP/Cy5.5) at a nanoparticle concentration of 50 µg/mL (containing 5 µg/mL Cy5.5, 10% w/w) were added to the upper chamber. The Transwell inserts were then transferred to the lower chamber containing PC12 cells (cultured in complete medium), and co‑incubated for 4 h. After incubation, PC12 cells in the lower chamber were fixed and imaged using a fluorescence microscope to assess the amount of Cy5.5 that crossed the BBB model.

### 4.27 MCAO Mouse Model

Transient focal cerebral ischemia was induced in female C57BL/6 mice using the intraluminal filament method. Mice were anesthetized with 2.5% tribromoethanol via intraperitoneal injection at a dose of 0.2 mL/10 g body weight. Body temperature was maintained at 37 ± 0.5°C using a heating pad during surgery. The mouse was placed in a supine position, and a midline incision was made in the neck. The right common carotid artery (CCA), external carotid artery (ECA) and internal carotid artery (ICA) were carefully isolated under a dissecting microscope. The CCA was ligated with a 6–0 silk suture near the heart, and the ECA was ligated at its distal end. A small incision was made in the CCA just proximal to the bifurcation, and a silicone‐coated nylon filament (diameter 0.18 mm, length 20 mm) was inserted into the ICA through the incision. The filament was advanced approximately 9–10 mm from the bifurcation until mild resistance was felt, indicating occlusion of the middle cerebral artery origin. After 1 h of ischemia, the filament was gently withdrawn to allow reperfusion. The incision was sutured, and the mice were allowed to recover in a warm cage. Sham‐operated mice underwent the same surgical procedure without filament insertion.

### 4.28 In Vivo Targeting Capability

One hour after reperfusion, MCAO mice (*n* = 3 per group) received tail vein injections of either free Cy5.5 or Cy5.5‑labeled nanoparticles (HMPB/Cy5.5, M@HP/Cy5.5, or RM@HP/Cy5.5). Free Cy5.5 was administered at a dose of 20 µg per mouse (in 200 µL PBS). For nanoparticle groups, each mouse received 10 mg/kg (based on nanoparticle mass, in 200 µL PBS), corresponding to a Cy5.5 dose of 20 µg per mouse (10% w/w loading), ensuring equal Cy5.5 fluorescence among all groups. Whole‑body fluorescence images were acquired using an in vivo imaging system (IVIS) at 4 h and 24 h post‑injection to assess targeting efficiency and biodistribution. At 24 h post‑injection, mice were euthanized, and major organs (heart, liver, spleen, lungs, kidneys, and brain) were harvested for ex vivo imaging. Fluorescence signals were quantified using Living Image software.

### 4.29 In Vivo Treatment Groups

MCAO mice were randomly divided into six groups (*n* = 3 per group): sham surgery group, saline group, HMPB group, HPAN group, M@HPAN group, and RM@HPAN group. One hour after reperfusion, mice received the first tail vein injection of the corresponding formulation (saline or nanoparticles at 10 mg/kg in 200 µL PBS). Subsequent injections were administered every other day for 14 days (total of 8 injections: 2 h, and days 2, 4, 6, 8, 10, 12, and 14 post‑MCAO). Mice were euthanized on day 14 after the final treatment. Brains were then collected, dehydrated at 4°C using sucrose solutions of varying concentrations, and sectioned. Brain sections were stained using a TUNEL apoptosis detection kit. Neurons within the brain were stained using Nissl staining. Oxidative stress (ROS) levels in brain tissue cells were detected via flow cytometry.

### 4.30 TTC Staining

After the final treatment, mice were euthanized, and brains were rapidly removed and frozen at −20 °C for 10 min. Coronal sections of 2 mm thickness were cut using a cryostat. The sections were then incubated in 2% (w/v) TTC solution in PBS at 37 °C for 30 min in the dark, until the viable tissue stained deep red and the infarct area remained pale white. After staining, sections were fixed in 4% paraformaldehyde overnight. Images were captured using camera, and the infarct area was quantified using ImageJ software.

### 4.31 7T T2WI MRI and Infarct Quantification

Mice underwent coronal T2WI scanning on a Bruker 7T Biospec MRI system with a mouse 1H surface coil under continuous isoflurane anesthesia. RARE sequence parameters: TR = 3000 ms, TEeff = 36 ms, matrix 256 × 256, FOV 20 × 20 mm, slice thickness 1 mm, 12 contiguous brain slices. Images were exported as TIFF stacks from ParaVision 5.1. Offline volumetric analysis was performed in ImageJ by a blind researcher. RGB images were converted to 8‐bit grayscale, and identical brightness/contrast window levels were applied to all samples. Spatial calibration (256 pixels = 20 mm, global) was implemented before manual polygon tracing of infarct, ipsilateral and contralateral brain regions slice‐by‐slice. Raw infarct volume = total summed infarct area × 1 mm slice thickness.

### 4.32 mNSS Behavioral Assessment

Neurological function was evaluated using the modified neurological severity score (mNSS) at day 0 (pre‑surgery), day 7, and day 14 post‑MCAO (or post‑reperfusion) by an investigator blinded to the treatment groups. The mNSS comprises four domains: motor tests (tail‑lift test and flat‑ground walking), balance beam test, sensory tests (placing and proprioceptive tests), and reflex absence. Each test yields a score, and the total mNSS ranges from 0 to 14 (0 = normal, 14 = maximal deficit).

### 4.33 Cylinder Test

Forelimb use asymmetry was assessed using the cylinder test at day 14 post‑MCAO by an investigator blinded to the treatment groups. Each mouse was placed individually into a transparent acrylic cylinder (height: 11 cm, diameter: 10 cm) with a mirror placed behind to allow 360° observation. The mouse's forelimb contacts with the cylinder wall during spontaneous vertical exploration were recorded using a video camera for 5 min per session. The number of left (ipsilateral) forelimb contacts, right (contralateral) forelimb contacts, and both forelimbs simultaneously (double contacts) was counted offline. The asymmetry score was calculated as: Asymmetry score (%) = (Left contacts—Right contacts) / (Left contacts + Right contacts + Double contacts) × 100. A higher asymmetry score indicates poorer motor coordination, reflecting a greater compensatory reliance on the ipsilateral (unaffected) forelimb.

### 4.34 Inflammatory Cytokine Expression

Brain tissue from the ischemic hemisphere was homogenized in RIPA lysis buffer containing protease inhibitors. After centrifugation at 2,000 × g for 10 min at 4 °C, the supernatant was collected. Levels of IL‑10, TGF‑β1, IL‑6, and TNF‑α were measured using commercial ELISA kits according to the manufacturers’ protocols. Absorbance was read at 450 nm, and cytokine concentrations were calculated from standard curves.

### 4.35 Western Blot Analysis

Protein expression levels were assessed by Western blot. Brain tissue samples or cultured cells were lysed in RIPA buffer containing 1% protease inhibitor cocktail. Protein concentration was determined using a BCA assay kit. Equal amounts of protein (30 µg per lane) were separated by 10%–12% SDS‐PAGE and transferred onto polyvinylidene fluoride (PVDF) membranes. Membranes were blocked with 5% non‐fat milk in TBST (Tris‐buffered saline with 0.1% Tween‐20) for 1 h at room temperature, then incubated overnight at 4°C with primary antibodies against the following targets: CD47 (Proteintech, 1:1000), Band 3 (Proteintech, 1:500), CHRNA7 (Abcam, 1:1000), Rap1 (Affinity, 1:1000), Rap1‐GTP (Affinity, 1:500), PI3K (Affinity, 1:1000), p‐PI3K (Invitrogen, 1:1000), Akt (Abcam, 1:1000), p‐Akt (Bioss, 1:1000), Bcl‐2 (Bioss, 1:1000), Bax (Proteintech, 1:1000), cleaved caspase‐3 (CST, 1:1000), p‐NF‐κB p65 (Affinity, 1:1000), Nrf2 (ZEN BIO, 1:1000), HO‐1 (ZEN BIO, 1:1000), BVR‐B (Huamei, 1:1000), β‐actin (Affinity, 1:2000), GAPDH (Proteintech, 1:2000), and Lamin B (Boster Bioengineering, 1:1000). After washing, membranes were incubated with HRP‐conjugated secondary antibodies (Beyotime, 1:5000) for 1 h at room temperature. Protein bands were visualized using an enhanced chemiluminescence (ECL) kit and imaged with a ChemiDoc MP system (Bio‐Rad). Band intensities were quantified using ImageJ software and normalized to the loading control (β‐actin, GAPDH, or Lamin B).

### 4.36 Nissl Staining

Brain tissues were fixed in 4% paraformaldehyde, dehydrated in graded sucrose solutions, and embedded in optimal cutting temperature (OCT) compound. Coronal sections (10 µm thick) were cut using a cryostat (Leica, Germany). Sections were washed with PBS, stained with 0.1% cresyl violet solution (Beyotime) for 10 min at 37 °C, then differentiated in 95% ethanol for 30 s. After dehydration in absolute ethanol and clearing in xylene, sections were mounted with neutral resin and observed under a light microscope (Olympus, Japan). The number of Nissl bodies was quantified in the ischemic penumbra region.

### 4.37 TUNEL Staining

Apoptotic cells in brain tissue sections were detected using a TUNEL assay kit according to the manufacturer's instructions. Briefly, frozen brain sections (10 µm) were fixed with 4% paraformaldehyde for 20 min, permeabilized with 0.1% Triton X‑100 in 0.1% sodium citrate for 2 min on ice, and then incubated with the TUNEL reaction mixture for 1 h at 37 °C in a humidified dark chamber. After washing with PBS, sections were counterstained with DAPI. Images were captured using a fluorescence microscope (Olympus), and the percentage of TUNEL‑positive cells was calculated from at least three randomly selected fields per section.

### 4.38 Determination of ROS Levels in Brain Tissue

ROS levels in brain tissue were measured using the DCFH‑DA probe. Brain tissues from the ischemic hemisphere were harvested, minced, and digested with 0.25% trypsin‑EDTA for 15 min at 37 °C to obtain a single‑cell suspension. Cells were washed twice with PBS, then incubated with 10 µM DCFH‑DA in serum‑free medium for 30 min at 37 °C in the dark. After washing, fluorescence intensity was measured using a flow cytometer (CytoFLEX) at excitation/emission wavelengths of 488/525 nm. Data were analyzed with FlowJo software.

### 4.39 Measurement of NO Levels in Brain Tissue

NO levels in brain tissue homogenates were measured using the Griess method. Brain tissue from the ischemic hemisphere was homogenized in PBS, and the supernatant was collected after centrifugation at 12,000 × g for 10 min at 4 °C. The NO content was determined using a nitric oxide assay kit following the manufacturer's protocol. Briefly, 50 µL of sample supernatant was mixed with 50 µL of Griess Reagent I and 50 µL of Griess Reagent II, incubated at room temperature for 10 min, and the absorbance at 540 nm was measured using a microplate reader (SpectraMax i3x). NO concentration was calculated from a standard curve generated with sodium nitrite.

### 4.40 Transcriptomics and Proteomics

Brain tissues from the model and RM@HPAN groups (*n* = 3) were subjected to RNA‑seq and LC‑MS/MS analysis. Differentially expressed genes (DEGs) and proteins (DEPs) were identified using |log_2_FC| > 1 and *p* < 0.05 as cutoffs.

### 4.41 In Vivo Biocompatibility Evaluation

Healthy female C57BL/6 mice were divided into two groups (*n* = 3 per group). Mice received tail vein injections of either saline or RM@HPAN (10 mg/kg in 200 µL PBS) every other day for 14 days. Body weight was recorded throughout the study. After the final injection, mice were euthanized, and major organs were harvested for hematoxylin and eosin (H&E) staining. Routine blood biochemical parameters were also assessed.

### 4.42 Statistical Analysis

All data were analyzed using GraphPad Prism software (version 10.0) and expressed as mean ± standard deviation (SD). Comparisons between two groups were performed using two‑tailed Student's *t*‑test. For multiple group comparisons, one‑way analysis of variance (ANOVA) was used, followed by Tukey's post hoc test for pairwise comparisons. A *p*‑value <0.05 was considered statistically significant. Significance levels are indicated as: *p < 0.05, **p < 0.01, ***p < 0.001, ****p < 0.0001; ns indicates not significant.

## Author Contributions

**Shaofa Li**: conceptualization, methodology, investigation, data curation, writing – original draft, funding acquisition. **Ying Wu**: investigation, data interpretation. **Huimin Wei**: formal analysis. **Jinyong Teng**: investigation. **Zaizhi Yang**: investigation. **Jufang Chen**: investigation, data curation. **Bao Liao**: methodology. **Xiaoqin Huang**: methodology, validation. **Lanqing Meng**: resources, writing – review & editing. **Tong Li**: supervision, writing – review & Editing. The manuscript has been read and approved by all co‐authors.

## Conflicts of Interest

The authors declare no conflict of interest.

## Supporting information




**Supporting File**: adhm71712‐sup‐0001‐SuppMat.docx.

## Data Availability

The data that support the findings of this study are available from the corresponding author upon reasonable request.
