# Supplementary material for: An Engineered Biomimetic Nanoreactor With Cascade Functions for Targeted and Combinatorial Therapy of Ischemic Stroke
Source: Adv Healthc Mater. 2026 Sep 13;15(38):e71712. doi: 10.1002/adhm.71712 (PMC13674122; doi:10.1002/adhm.71712)
Supplement: Supplementary file 1 [file ADHM-15-0-s001.docx]

**Supporting Information**

**An Engineered Biomimetic Nanoreactor with Cascade Functions for Targeted and Combinatorial Therapy of Ischemic Stroke**

*Shaofa Li^1, *^, Ying Wu^1^, Huimin Wei^1^, Jinyong Teng^1^, Zaizhi Yang^1^, Jufang Chen^1^, Bao Liao^1^, Xiaoqin Huang^1^, Lanqing Meng^2, *^, Tong Li^3, *^*

^1^Youjiang Medical University for Nationalities Affiliated Southwest Hospital, Baise People's Hospital, Guangxi, China.

^2^Department of Neurology, Affiliated Hospital of Youjiang Medical University for Nationalities, Guangxi, China.

^3^Nanning Second People's Hospital Neurology Department, Guangxi, China.

**^*^Correspondence:** Shaofa Li (13977693812@ymun.edu.cn); Lanqing Meng (mlq1864@ymun.edu.cn); Tong Li (qt000182@sr.gxmu.edu.cn)

**Figure S1.** (A) solution photograph and (B) UV-vis spectrum of HMPB.

**Table S1.** Drug loading and encapsulation efficiency of the two drugs in HPAN.

| Sample | Drug | EE% | DL% |
| --- | --- | --- | --- |
| HPAN | Arg | 42.8 ± 1.2 | 21.4 ± 0.6 |
|  | NBP | 55.4 ± 0.8 | 2.8 ± 0.04 |

**Figure S2.** SDS-PAGE protein analysis. Ⅰ: HMPB, Ⅱ: HPAN, Ⅲ: Red blood cell membrane, Ⅳ: M@HPAN, Ⅴ: RM@HPAN.

**Figure S3.** Fluorescence microscopy images of HPAN NPs and Red blood cell membrane NPs (green: Red blood cell membrane NPs; red: HPAN NPs; scale bar: 5 μm).

**Figure S4.** (A) Arg release profiles of HPAN and RM@HPAN under neutral (pH7.4) and acidic conditions (pH5.0). (B) NBP release profiles of HPAN and RM@HPAN under neutral (pH7.4) and acidic conditions (pH5.0). Data were expressed as mean ± SD (n=3).

**Figure S5.** Cell viability after 24 h incubation with (A) bEnd.3 and (B) PC12 cells at various concentrations of RM@HPAN. Data were expressed as mean ± SD (n=3).

**Figure S6.** Cellular uptake of different nanoparticles in RAW264.7 macrophages after CD47 blockade (scale bar: 20 μm). Data were expressed as mean ± SD (n=3). n.s. No significant. Data were analyzed by one-way ANOVA with Tukey's post hoc test.

**Figure S7.** Uptake of different samples by bEnd.3 (scale bar: 20 μm) and corresponding fluorescence intensity quantification. Data were expressed as mean ± SD (n=3). *P<0.05, **P<0.01, n.s. No significant. Data were analyzed by one-way ANOVA with Tukey's post hoc test.

**Figure S8.** NO fluorescence images of control, LPS, LPS+Arg, and LPS+RM@HPAN after 4 h of incubation with bEnd.3, respectively (scale bar: 20 μm) and corresponding relative fluorescence quantification. Data were expressed as mean ± SD (n=3). ****P<0.0001, n.s. No significant. Data were analyzed by one-way ANOVA with Tukey's post hoc test.

**Figure S9.** (A) Flow cytometry was used to detect the effect of different samples on the apoptosis of PC12 cells and (B) the quantitative plot of apoptosis rate. (C) Cell viability of different nanoparticle-treated PC12 cells in the OGD/R model. Data were expressed as mean ± SD (n=3). **P<0.01, ****P<0.0001, n.s. No significant. Data were analyzed by one-way ANOVA with Tukey's post hoc test.

**Figure S10.** DCFH-DA (ROS probe) fluorescence images of control, OGD/R, HMPB, HPAN, M@HPAN and RM@HPAN after 24 h of incubation with PC12, respectively (scale bar: 20 μm) and corresponding relative fluorescence quantification. Data were expressed as mean ± SD (n=3). ***P<0.001, ****P<0.0001, n.s. No significant. Data were analyzed by one-way ANOVA with Tukey's post hoc test.

**Figure S11.** Transepithelial electrical resistance of different sample groups. Data were expressed as mean ± SD (n=3). n.s. No significant. Data were analyzed by one-way ANOVA with Tukey's post hoc test.

**Figure S12.** (A) Representative T2-weighted MRI images of the brain in mice from the sham surgery group and the MCAO group following different treatments, and (B) quantitative analysis of infarct volume. Data were expressed as mean ± SD (n=3). *P<0.05, **P<0.01, ***P<0.001, ****P<0.0001, Data were analyzed by one-way ANOVA with Tukey's post hoc test.

**Figure S13.** Determination of NO levels in the brain after different treatments using the Griess method. Data were expressed as mean ± SD (n=3). ****P<0.0001, Data were analyzed by one-way ANOVA with Tukey's post hoc test.

**Figure S14.** Hemolytic rate test results of the RM@HPAN nanoparticles. Data were expressed as mean ± SD (n=3).

**Figure S15.** (A) Evaluation of CREA, (B) AST and (C) ALT after treatment with saline or RM@HPAN. Data were expressed as mean ± SD (n=3). n.s. No significant, Data were analyzed using Student’s t-test.

**Figure S16.** (A) Evaluation of body weight and (B) organ index of mice after treatment with saline or RM@HPAN. Data were expressed as mean ± SD (n=3).

**Figure S17.** Typical images of H&E staining of main organs in RM@HPAN group and saline group mice (scale bar: 50 μm) (n=3).

**Table S2.** Core functions and validation methods of each component in RM@HPAN.

| **Component** | **Core Functions** | **Validation Experiments & Key Results** | **Experimental Methods** |
| --- | --- | --- | --- |
| Hollow Mesoporous Prussian Blue (HMPB) | 1. ROS scavenging (multi-enzyme mimic activity: SOD-like, CAT-like);  2. Drug (L-arginine/NBP) loading scaffold | 1. In vitro ROS scavenging assay: RM@HPAN reduced OGD/R-induced ROS by 84.3% (vs. OGD/R control, ****P<0.0001);  2. Comparison with commercial antioxidants (SOD, CAT): RM@HPAN exhibited 1.8-fold higher ROS clearance efficiency (Supplementary Figure S10);  3. Drug loading efficiency: L-arginine (21.4%), NBP (2.8%) | 1. DCFH-DA fluorescent probe for ROS detection;  2. SOD/CAT activity kits;  3. UV-vis for drug loading quantification |
| L-arginine (Arg) | 1. NO production via NOS catalysis;  2. Synergistic neuroprotection with ROS scavenging | 1. L-NAME inhibition assay: NO synthase inhibitor reversed 12.2% of RM@HPAN’s cell viability enhancement (**P<0.01, Supplementary Figure S9);  2. In vivo NO detection: RM@HPAN increased ischemic brain NO/nitrite/nitrate by 2.8-fold (vs. Saline, Supplementary Figure S13) | 1. OGD/R model + L-NAME (1 mM) treatment;  2. Griess reaction kit for NO metabolite detection |
| Butylphthalide (NBP) | 1. Improvement of cerebral blood flow;  2. Inhibition of neuronal apoptosis;  3. Synergistic enhancement with HMPB/L-arginine | Head-to-head comparison (RM@HPAN vs. RM@HP, lacking NBP):  -Cell viability: 22% higher in RM@HPAN (****P<0.0001);  -Apoptosis rate: 39.4% lower in RM@HPAN (****P<0.0001, Supplementary Figure S9) | OGD/R model with PC12 cells;  CCK-8 (viability), TUNEL (apoptosis) |
| Red Blood Cell (RBC) Membrane | 1. Immune evasion (CD47-SIRPα pathway);  2. Prolonged systemic circulation | 1. Macrophage phagocytosis assay: M@HPAN/RM@HPAN uptake by RAW264.7 cells reduced by 50.9%/77.1% (vs. HPAN, **P<0.01, Figure 3A-B);  2. CD47 blocking assay: Anti-CD47 antibody reversed phagocytosis inhibition (P>0.05 vs. HPAN, Supplementary Figure S6);  3. In vivo biodistribution: Liver accumulation reduced by 26.6% (vs. HPAN, *P<0.05) | 1. Fluorescence imaging (phagocytosis);  2. Anti-CD47 neutralizing antibody (5 μg/mL) pre-treatment;  3. IVIS imaging (biodistribution) |
| RVG-29 Peptide | BBB penetration (nAChR-mediated transcytosis) | 1. Inflammation-induced endothelial uptake assay: RM@HPAN uptake by inflammatory bEnd.3 was 2.0‑fold higher than M@HPAN and 4.4‑fold higher than HPAN (**P<0.01, Figure 3D,E);  2. In vitro blood-brain barrier model: The amount of RM@HPAN crossing the BBB and taken up by PC12 neurons was 3.9‑fold higher than M@HPAN and 4.5‑fold higher than HPAN (****P < 0.0001, Figure 4A-C) | 1. bEnd.3 cell uptake assay;  2. Co-culture BBB model (bEnd.3 + neurons) |

**Table S3.** Biodistribution comparison of RM@HPAN with recent RVG-29 targeted nanosystems for ischemic stroke.

| **Nanoparticle System** | **RVG-29 Mediated Brain Enrichment (Fold Change vs. Non-targeted Control)** | **Liver Non-targeted Distribution (Fold Change vs. Non-targeted Control)** | **Reference** |
| --- | --- | --- | --- |
| RVG-EVs | 1.3 | 0.75 | [1] |
| RPSeLipo | 1.9 | -- | [2] |
| T-LNPs | 1.3 | -- | [3] |
| DiD-Pue^Lipo^/R-R | 3.4 | -- | [4] |
| RVG29-PNPs@si-IRF9 | 2.0 | 0.7 | [5] |
| RM@HPAN (Our system) | 6.0 | 0.18 | This study |

**References**

1. S. Guo, Z. Wang, R. Shi, et al., “Engineered Extracellular Vesicles for Targeted FGF20 Delivery Enhance Neuroplasticity and Functional Recovery in Ischemic Stroke,” *Journal of Controlled Release*, (2025): 114080, <https://doi.org/10.1016/j.jconrel.2025.114080>
2. S. Tian, Q. Zhao, Z. Liu, et al., “The Biofunctional Nanomaterials of Iron-Chelating and Antioxidant Targeting the Blood-Brain Barrier in the Injured Area Alleviated Cerebral Ischemia-Reperfusion Injury,” *Nano Today*, (2025): 102874, <https://doi.org/10.1016/j.nantod.2025.102874>
3. W. Shi, S. Yuan, G. Cheng, et al., “Blood Brain Barrier-Targeted Lipid Nanoparticles Improved the Neuroprotection of Ferrostatin-1 Against Cerebral Ischemic Damage in An Experimental Stroke Model,” *Experimental Neurology*, (2024): 114849, <https://doi.org/10.1016/j.expneurol.2024.114849>
4. D. Chen, H. Jiang, L. Sun, et al., “Neuron-Targeted ROS-Responsive Liposomes for Puerarin Delivery Remodel Ischemic Microenvironment via Microglial Modulation and Neurovascular Regeneration,” *Journal of Nanobiotechnology*, (2025): 677, <https://doi.org/10.1186/s12951-025-03730-2>
5. F. Zhang, W. Shen, S. Zhong, et al., “Therapeutic Targeting of the IRF9/RTN4/RHOA/ROCK Pathway via RVG29-Modified PLGA Nanoparticles and rTMS for Neural and Vascular Regeneration Post-Cerebral Infarction,” *Advanced Healthcare Materials*, (2026): e01846, https://doi.org/10.1002/adhm.202501846
